# Lefamulin Overcomes Acquired Drug Resistance via Regulating Mitochondrial Homeostasis by Targeting ILF3 in Hepatocellular Carcinoma

**DOI:** 10.1002/advs.202401789

**Published:** 2024-06-14

**Authors:** Ying Zheng, Shengtao Ye, Shiyu Huang, Yang Cheng, Yanqiu Zhang, Yingrong Leng, Mengmeng He, Enyi Wu, Junxin Chen, Lingyi Kong, Hao Zhang

**Affiliations:** ^1^ Jiangsu Key Laboratory of Bioactive Natural Product Research and State Key Laboratory of Natural Medicines School of Traditional Chinese Pharmacy China Pharmaceutical University Nanjing 210009 China

**Keywords:** acetylation, HCC, lefamulin, ILF3, MRPL12, targeted therapy resistance

## Abstract

Acquired resistance represents a critical clinical challenge to molecular targeted therapies such as tyrosine kinase inhibitors (TKIs) treatment in hepatocellular carcinoma (HCC). Therefore, it is urgent to explore new mechanisms and therapeutics that can overcome or delay resistance. Here, a US Food and Drug Administration (FDA)‐approved pleuromutilin antibiotic is identified that overcomes sorafenib resistance in HCC cell lines, cell line‐derived xenograft (CDX) and hydrodynamic injection mouse models. It is demonstrated that lefamulin targets interleukin enhancer‐binding factor 3 (ILF3) to increase the sorafenib susceptibility of HCC via impairing mitochondrial function. Mechanistically, lefamulin directly binds to the Alanine‐99 site of ILF3 protein and interferes with acetyltransferase general control non‐depressible 5 (GCN5) and CREB binding protein (CBP) mediated acetylation of Lysine‐100 site, which disrupts the ILF3‐mediated transcription of mitochondrial ribosomal protein L12 (MRPL12) and subsequent mitochondrial biogenesis. Clinical data further confirm that high ILF3 or MRPL12 expression is associated with poor survival and targeted therapy efficacy in HCC. Conclusively, this findings suggest that ILF3 is a potential therapeutic target for overcoming resistance to TKIs, and lefamulin may be a novel combination therapy strategy for HCC treatment with sorafenib and regorafenib.

## Graphical abstract

Lefamulin overcomes drug resistance of hepatocellular carcinoma (HCC) by targeting interleukin enhancer‐binding factor 3 (ILF3) and interfering with general control non‐depressible 5 (GCN5) and CREB binding protein (CBP)‐mediated acetylation, which inhibits mitochondrial ribosomal protein L12 (MRPL12) transcription and regulates mitochondrial homeostasis. Lefamulin may be a novel combination therapy strategy for HCC treatment with tyrosine kinase inhibitors (TKIs).

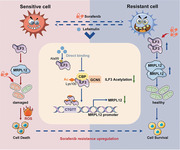

## 1 Introduction

Liver cancer is the fourth leading cause of cancer‐related death worldwide, hepatocellular carcinoma (HCC) is the dominant histological type of liver cancer and accounts for approximately 90% of liver cancer cases.^[^ ^1^ ^]^ Sorafenib is a multi‐kinase inhibitor approved in 2007 as the first systemic drug to treat advanced unresectable HCC.^[^ ^2^ ^]^ Since then, regorafenib has been approved for the second‐line treatment of advanced HCC, and lenvatinib was found to be noninferior to sorafenib as a first‐line agent.^[^ ^3^ ^]^ Although advances in immunotherapy for HCC have also added hope for patients, their efficacy remains limited. Sorafenib remains a cornerstone treatment in HCC that is supported by robust evidence and clinical experience.^[^ ^4^ ^]^ Unfortunately, only approximately 30% of patients can benefit from sorafenib, and this population usually acquires drug resistance within 6 months.^[^ ^5^ ^]^ However, the mechanisms underlying resistance have not been fully clarified and remain poorly understood. Therefore, it is urgent to find new therapeutic targets and agents to overcome sorafenib resistance.

Pleuromutilins, a class of antibiotics derived from the fungal diterpene, clinically used against Gram‐positive pathogens. Tiamulin was the first pleuromutilin compound approved for veterinary use, followed by valnemulin. Retapamulin was the first pleuromutilin approved for skin infections caused by susceptible streptococci or staphylococci in humans.^[^ ^6^ ^]^ However, retapamulin is limited to topical application. Lefamulin is a novel oral and intravenous (IV) pleuromutilin for the treatment of community‐acquired bacterial pneumonia (CABP).^[^ ^7^ ^]^ Up to date, there are no reports that lefamulin alone or in combination with other agents possess considerable antitumor properties. Therefore, a deeper understanding of the potential antitumor activity and mechanisms of lefamulin provides insights into cancer science and helps to develop novel therapeutic strategies.

Mitochondrial metabolism affects all steps related to tumor development by generating building blocks of tumor synthesis metabolism, maintaining redox homeostasis, participating in transcriptional regulation, and controlling cell death.^[^ ^8^ ^]^ Studies have shown that drug‐resistant tumor cells have increased mitochondrial biogenesis,^[^ ^9^ ^]^ and depend on mitochondrial biogenesis and respiration to increase susceptibility to mitochondrial targeted drugs.^[^ ^10^, ^11^ ^]^ Mitochondrial ribosomal proteins (MRPs) are important components of the structural and functional integrity of mitochondrial ribosomal complexes. Mitochondrial ribosomal protein L12 (MRPL12) belongs to the MRPs, functions in mitochondrial respiration, cell viability, growth and differentiation.^[^ ^12^ ^]^ MRPL12 is required for CycD/Cdk4‐induced cell growth, and MRPL12 mutant cells exhibit reduced mitochondrial activity and growth defects.^[^ ^13^ ^]^ MRPL12 interacts with the mitochondrial RNA polymerase POLRMT to regulate mitochondrial transcription, translation and/or ribosomal biogenesis and mitochondrial gene expression.^[^ ^14^ ^]^ MRPL12 has also been identified as a risk factor and independent prognostic factor for breast cancer.^[^ ^15^ ^]^ However, the role of MRPL12 in HCC and sorafenib resistance remain poorly understood.

Interleukin enhancer‐binding factor 3 (ILF3), also known as NF90/NF110, is a double stranded RNA binding protein (DRBP).^[^ ^16^ ^]^ ILF3 is involved in various cellular functions, such as mRNA stabilization, non‐coding RNA biogenesis and translation inhibition by binding to different cellular RNAs.^[^ ^17^ ^]^ Previous studies demonstrated that ILF3 was overexpressed in various cancers, and positively correlated with poor survival of patients.^[^ ^18^ ^]^ ILF3 could regulate the stability of VEGF mRNA and promote breast cancer growth,^[^ ^19^ ^]^ as well as the stability of cyclin E1 mRNA and participate in the regulation of liver cancer cell cycle.^[^ ^20^ ^]^ However, the role of ILF3 in sorafenib resistance in HCC is unclear to date.

Here, we identified pleuromutilin antibiotics including retapamulin, valnemulin and lefamulin as candidates for sorafenib‐sensitizing drugs using a functional screening from a library comprising 1430 U.S. Food and Drug Administration (FDA)‐approved drugs. Lefamulin targets the Alanine‐99 site of ILF3 protein and interferes with acetyltransferase general control non‐depressible 5 (GCN5) and CREB binding protein (CBP) mediated acetylation of Lysine‐100 site, suppressing the ILF3‐mediated transcription of MRPL12 and subsequent mitochondrial biogenesis. Lefamulin was well tolerated in mice and not only directly inhibited tumor progression but also overcame drug resistance and potentiated the antitumor effect of sorafenib and regorafenib in cell line‐derived xenograft (CDX) and hydrodynamic injection mouse models. These findings may provide a novel mechanism involving ILF3‐MRPL12 axis in sorafenib resistance, and a promising combination strategy of lefamulin and targeted therapy for HCC treatment.

## 2 Results

### 2.1 Identification of Pleuromutilin Class of Antibiotics as Sensitizers for Sorafenib from FDA‐Approved Library

**Figure 1:**
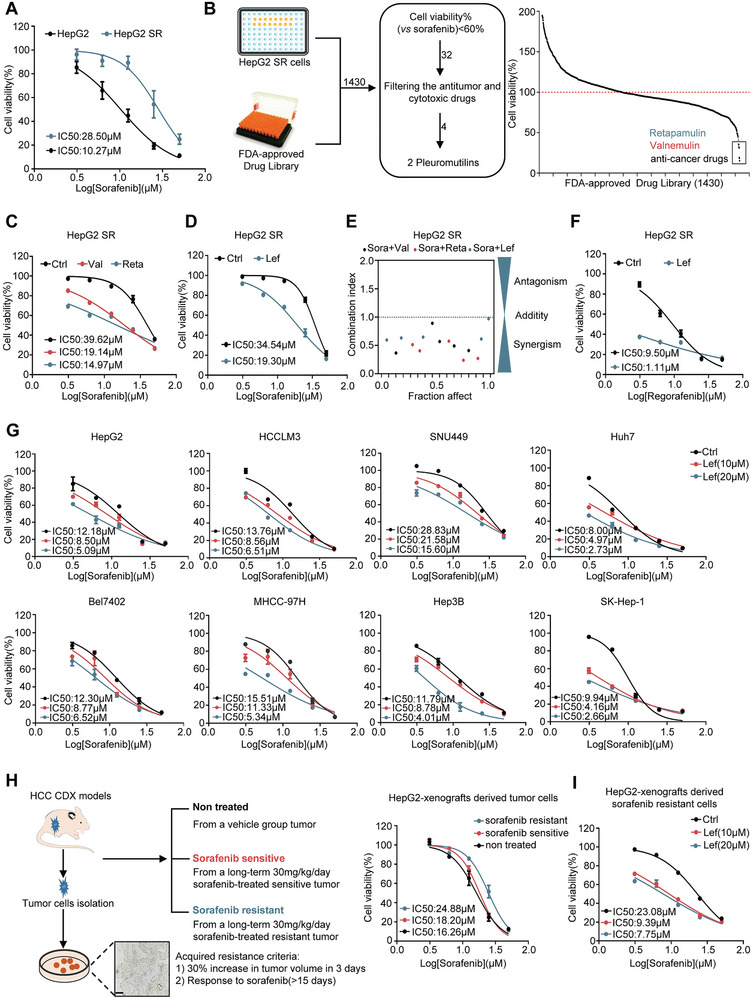
Identification of pleuromutilin class of antibiotics as sensitizers for sorafenib from FDA‐approved library. A) The viability of sorafenib‐resistant (HepG2 SR) cells and its corresponding parental cells HepG2 cells were analyzed by CCK‐8 assay. Cells were treated with various concentrations of sorafenib for 48 h (*n* = 3). B) Left, screening diagram showing the procedure for screening drugs in FDA‐approved drug library that can enhance the sensitivity of sorafenib. Right, statistical distribution curve of cell viability in HCC cells of 1430 FDA‐approved drugs. C,D) HepG2 SR cells were treated with various concentrations of sorafenib alone, or in combination with 10 × 10^−6^ m C) valnemulin (val), retapamulin (reta) or D) lefamulin (lef) for 48 h, and cell viability was evaluated by CCK‐8 assay (*n* = 3). E) Synergistic effect of valnemulin, retapamulin or lefamulin with sorafenib on HepG2 SR cells. CI (combination index) values were calculated as described in Supporting Information. F) HepG2 SR cells were treated with various concentrations of regorafenib alone, or in combination with 10 × 10^−6^ m lefamulin for 48 h, and cell viability was evaluated by CCK‐8 assay (*n* = 3). G) Cell viability assay was carried out in different HCC cell lines, including HepG2, HCCLM3, SNU449, Huh7, Bel7402, MHCC‐97H, Hep3B and SK‐Hep‐1 cells treated with 10 × 10^−6^ or 20 × 10^−6^ m lefamulin and/or various concentrations of sorafenib for 48 h (*n* = 3). H) Left, experimental design of an HCC xenograft model of acquired resistance to sorafenib and the isolation and representative images of sorafenib‐resistant tumor cells. Scale bar, 5 µm. Right, HepG2‐xenografts derived tumor cells were treated with various concentrations of sorafenib for 48 h, and cell viability was analyzed by CCK‐8 assay (*n* = 3). I) HepG2‐xenografts derived sorafenib resistant cells were treated with various concentrations of sorafenib alone, or in combination with 10 × 10^−6^ or 20 × 10^−6^ m lefamulin for 48 h, and cell viability was evaluated by CCK‐8 assay (*n* = 3). Data are presented as mean ± SEM.

Drug repurposing is promoted as a cost‐ and time‐effective mechanism for providing new medicines. To explore potential drugs involved in overcoming sorafenib resistance in HCC, we first established two sorafenib‐resistant HCC cell lines by culturing cells with gradually increasing concentrations of sorafenib for approximately 6 months, named HepG2 SR and HCCLM3 SR cells (**Figure** **1**A; Figure S1A–D, Supporting Information). Subsequently, based on a drug library consisting of 1430 FDA‐approved compounds (Data file S1, Supporting Information), a combination treatment approach was applied to identify compounds with sensibilization properties to sorafenib in HepG2 SR cells. Relative cell viability was assessed after treatment with candidate drugs in the presence and absence of sorafenib, and we identified numerous compounds that showed synergistic effect with sorafenib (Table S1, Supporting Information). We found, excluding the anti‐cancer drugs, retapamulin and valnemulin significantly sensitized sorafenib in HepG2 SR cells, which attracted our attention (Figure 1B). Furthermore, we confirmed that retapamulin or valnemulin combined with sorafenib significantly inhibited cell growth and promoted apoptosis in HepG2 SR cells (Figure 1C; Figure S1E, Supporting Information), suggesting a synergistic effect of pleuromutilins and sorafenib treatment in resistant cell lines (Figure 1E). However, retapamulin and valnemulin have limitations respectively, retapamulin is for external use only, and valnemulin is for veterinary use only. Lefamulin, with the same maternal nuclear structure pleuromutilin, is the first oral human antibiotic for the treatment of community‐acquired bacterial pneumonia (CABP) (Figure S1F, Supporting Information). Cell viability assay revealed that the combination of lefamulin and sorafenib also showed a significant synergistic effect (Figure 1D,E; Figure S1G, Supporting Information). Moreover, lefamulin also improved the efficacy of regorafenib, a second‐line treatment for HCC patients who do not respond to sorafenib (Figure 1F; Figure S1H, Supporting Information). Next, we expanded this result to various HCC cell lines, and get a consistent synergistic effect (Figure 1G). However, compared to hepatoma cells, lefamulin showed no significant toxicity in human normal hepatocytes L02 cells (Figure S1I, Supporting Information). Simultaneously, we generated a sorafenib‐resistant xenograft mouse model of HCC in vivo, and isolated resistant cells from these tumors (Figure 1H). The drug resistance was confirmed by cell viability assay, resistance‐associated gene expression and immunohistochemical staining (IHC) (Figure 1H; Figure S1J,K, Supporting Information). As expected, lefamulin also increased the sensitivity of sorafenib and regorafenib in tumor cells isolated from drug resistant mice (Figure 1I; Figure S1L, Supporting Information). Collectively, as the first oral human used pleuromutilin antibiotic, lefamulin emerges as a promising reusable drug that can be used for a new indication, so we focus on the synergistic role of lefamulin on targeted therapy in HCC in the following.

### 2.2 Combination of Lefamulin and Sorafenib Significantly Inhibits HCC Growth In Vitro and In Vivo

**Figure 2:**
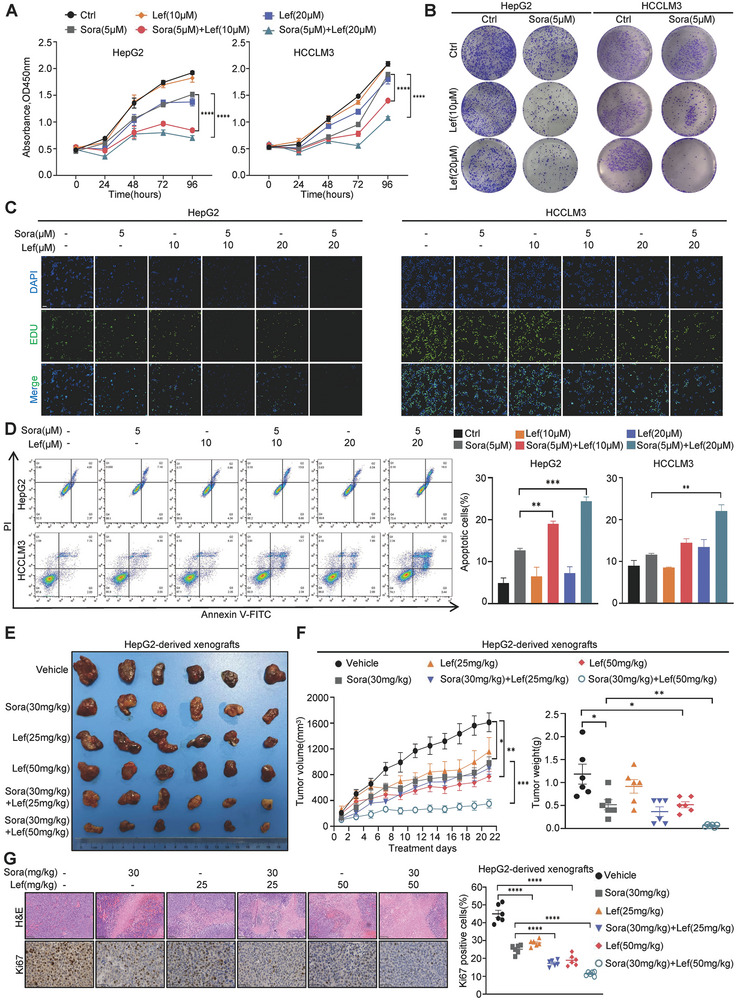
Combination of lefamulin and sorafenib significantly inhibits HCC growth in vitro and in vivo. A–C) HepG2 and HCCLM3 cells were treated with various concentrations of lefamulin and 5 × 10^−6^ m sorafenib separately or in combination for indicated time, and cell proliferation was evaluated by A) CCK‐8 assay (*n* = 3), B) colony formation assay and C) EDU incorporation assay (Scale bar, 100 µm). D) HepG2 and HCCLM3 cells were treated with various concentrations of lefamulin and 5 × 10^−6^ m sorafenib separately or in combination for 48 h, and apoptosis was analyzed by flow cytometry (*n* = 3). E–G) Nude mice bearing HepG2‐derived xenografts were intraperitoneally administered lefamulin (25 or 50 mg kg^−1^) and/or orally administered sorafenib (30 mg kg^−1^) every day, *n* = 6. Representative images of E) the tumors, F) tumor growth curves and tumor weight, G) representative images of hematoxylin and eosin (H & E) (Scale bar, 100 µm) and Ki67 staining (Scale bar, 50 µm) in tumor tissues are shown (*n* = 6). Data are presented as mean ± SEM. t test, *P < 0.05, **P < 0.01, ***P < 0.001, and ****P < 0.0001.

To further confirm the synergistic effect of lefamulin, cell growth curve, colony formation assay and EDU incorporation experiments were performed. We found that lefamulin alone or combined with sorafenib/regorafenib/lenvatinib significantly inhibited HCC cell proliferation (**Figure** **2**A–C; Figure S2A–F, Supporting Information). In addition, Annexin V‐propidium iodide (PI) assay was also performed to provide compelling evidence showing that lefamulin in combination with sorafenib significantly increased the apoptosis rate in HCC cells (Figure 2D). It is worth noting that lefamulin showed no obvious cell growth inhibitory effect on L02 cells (Figure S2G–I, Supporting Information). Given that sorafenib‐resistant HCC cells are endowed with enhanced cancer stem cell (CSC) properties,^[^ ^21^ ^]^ interestingly, we found lefamulin inhibited tumor‐initiating cell associated gene expression (Figure S2J, Supporting Information), which further supported our conclusion that lefamulin enhances the sensitivity of sorafenib in HCC.

To further examine the sensitization effect of lefamulin in vivo, we subcutaneously injected HepG2 cells into Balb/c nude mice, and randomly divided them into six groups receiving either vehicle control, lefamulin, sorafenib, or the combination treatment every day. The results showed that, compared to sorafenib treated group, the combination treated group significantly suppressed tumor growth as indicated by smaller tumor volume and decreased tumor weight, lefamulin alone also revealed moderate anti‐tumor effect (Figure 2E,F). The increased cell death and decreased Ki67 proliferation index further confirmed the synergistic effect of lefamulin on sorafenib (Figure 2G). Lefamulin was well tolerated in the dose range investigated, showing no serious treatment‐emergent adverse events indicated by body weight and pathological staining (Figure S2K,L, Supporting Information). Simultaneously, the plasma alanine aminotransferase (ALT), aspartate aminotransferase (AST), creatinine (CRE) and urea nitrogen (BUN) levels also did not significantly change (Figure S2M, Supporting Information). Collectively, these results indicate that lefamulin suppresses HCC progression in vitro and in vivo without obvious toxicity.

### 2.3 Lefamulin‐Mediated Mitochondrial Dysfunction Augmented the Susceptibility of HCC Cells to Sorafenib

**Figure 3:**
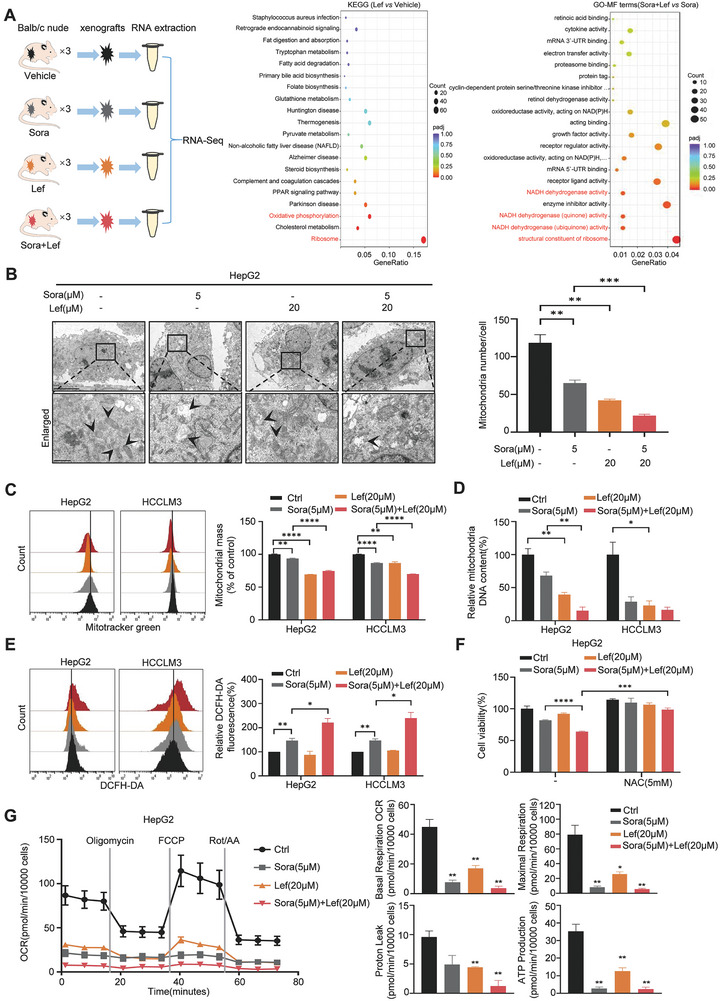
Lefamulin‐mediated mitochondrial dysfunction augmented the susceptibility of HCC cells to sorafenib. A) Left, scheme illustrating; middle, KEGG pathway analysis; right, Gene ontology (GO) analysis for differentially expressed genes of lefamulin and/or sorafenib treated tumors. B) Left, representative transmission electron microscopy (TEM) images of mitochondrial morphological changes in HepG2 cells after lefamulin and/or sorafenib treatment. Scale bar, 5 µm (top), 1 µm (bottom). Right, statistical chart of the numbers of mitochondria (*n* = 3). C) Mitochondrial mass was estimated with MitoTracker Green using flow cytometry in HepG2 and HCCLM3 cells treated with lefamulin and/or sorafenib (*n* = 3). D) RT‐qPCR analysis of mitochondrial DNA content in HepG2 and HCCLM3 cells treated with lefamulin and/or sorafenib (*n* = 3). E) Flow cytometric analysis of ROS accumulation using DCFH‐DA probe in HepG2 and HCCLM3 cells treated with lefamulin and/or sorafenib (*n* = 3). F) HepG2 cells were treated with 20 × 10^−6^ m lefamulin and/or 5 × 10^−6^ m sorafenib in the presence or absence of NAC (an ROS scavenger, 5 × 10^−6^ m) pre‐treatment for 24 h. The cell viability was determined by CCK‐8 assay (*n* = 3). G) Oxygen consumption rate (OCR) analysis using Seahorse analysis revealed compromised mitochondrial function in HepG2 cells treated with lefamulin and/or sorafenib for 48 h. Basal respiration, ATP production, maximal respiration, and spare respiratory capacity were identified, respectively (*n* = 3). Data are presented as mean ± SEM. t test, *P < 0.05, **P < 0.01, ***P < 0.001, and ****P < 0.0001.

To mechanistically investigate this synergism, RNA sequencing (RNA‐Seq) analysis was performed to get insight into the transcriptional profiles of tumors treated by vehicle control, lefamulin, sorafenib or the combination. KEGG pathway enrichment analysis and Gene Ontology (GO) analysis revealed that the mitochondrial‐related signal pathways were significantly altered upon lefamulin treatment (**Figure** **3**A). To ascertain the mitochondrial alterations in response to lefamulin treatment, we evaluated the morphology, quantity, and function of mitochondria. Morphological analysis of mitochondria with transmission electron microscopy (TEM) revealed mitochondrial swelling, mitochondrial cristae disintegration and fewer mitochondrial numbers in lefamulin, sorafenib and the combination treated groups, indicating worse mitochondrial integrity (Figure 3B). We then measured mitochondrial quantity by using the MitoTracker Green FM probe. After lefamulin, sorafenib or the combination treatment, a significant reduction in mitochondria mass was observed (Figure 3C). Consistently, mitochondrial DNA copy number, as well as the expression levels of mitochondrial components such as AIF, HSP60 and TOM20 were downregulated in lefamulin alone or combination treated cells compared with controls (Figure 3D; Figure S3A, Supporting Information). In addition, we found that lefamulin treatment increased mitochondrial ROS generation (Figure 3E). Cell viability assays revealed that lefamulin in combination with sorafenib induced tumor cell death was blocked by NAC (an ROS scavenger), partially blocked by Ferrostatin‐1 (a ferroptosis inhibitor) and Z‐VAD‐FMK (an apoptosis inhibitor), but not blocked by necrosulfonamide (a necroptosis inhibitor) (Figure 3F; Figure S3B, Supporting Information), suggesting the possible involvement of oxidative stress, apoptosis and ferroptosis pathway in the synergistic effects. To detect the oxidative phosphorylation level, the cell bioenergetic profiles were analyzed. In consistent with the RNA‐seq results, lefamulin treatment significantly inhibited basal oxygen consumption rate (OCR), ATP‐linked OCR, maximal respiration, and spare respiratory capacity, suggesting decreased oxidative phosphorylation level (Figure 3G). However, lefamulin showed no obvious effect on mitochondrial function of L02 and WRL68 cells (Figure S3C–G, Supporting Information). Thus, these results suggest that lefamulin impairs mitochondrial function to enhance the sensitivity of sorafenib in HCC cells.

### 2.4 Lefamulin Impairs Mitochondrial Function via Downregulation of MRPL12

**Figure 4:**
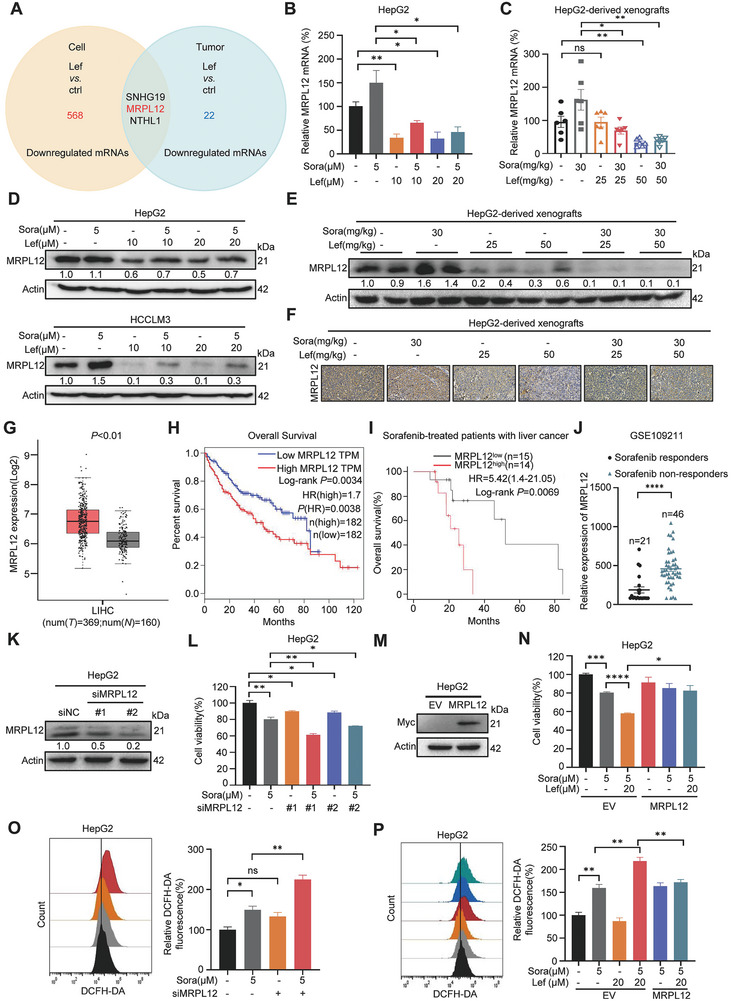
Lefamulin impairs mitochondrial function via downregulation of MRPL12. A) Venn diagram of the co‐downregulated mRNAs after lefamulin treatment in HepG2 cells and HepG2‐derived xenografts. B,C) RT‐qPCR was carried out to examine mRNA expression of *MRPL12* in B) HepG2 cells (*n* = 3) and C) HepG2‐derived xenografts (*n* = 6) treated with lefamulin and/or sorafenib. D,E) Western blot was carried out to examine protein expression of MRPL12 in D) HepG2 and HCCLM3 cells and E) HepG2‐derived xenografts treated with lefamulin and/or sorafenib. F) Representative images of IHC staining of MRPL12 of tumors from nude mice. Scale bar, 50 µm. G) The expression of MRPL12 in HCC (*n* = 369) and non‐tumor tissues (*n* = 160) (*P* < 0.01) from GEPIA database. Based on the selected TCGA tumors and TCGA normal + GTEx normal datasets performed difference analysis (one‐way ANOVA). H) Kaplan‐Meier overall survival (Log‐rank *P =* 0.0034) curves of HCC patients, using the TCGA datasets in the GEPIA database. High and low expression of MRPL12 were stratified by the median. I) Kaplan‐Meier analysis of overall survival (Log‐rank *P =* 0.0069) probability in liver cancer patients that have undergone sorafenib treatment. The patients were stratified according to high versus low expression (cutoff, median) of MRPL12 mRNA within their tumors. J) MRPL12 mRNA levels in tumors between sorafenib responders (*n* = 21) and sorafenib non‐responders (*n* = 46) from GEO database. K,M) Western blot was carried out to examine protein expression of MRPL12 in HepG2 cells after K) MRPL12 knockdown or M) overexpression. L,N) Effect of L) MRPL12 knockdown or N) overexpression on sorafenib sensitivity in HepG2 cells. The cells were treated for 48 h. Cell viability was measured by CCK‐8 assay (*n* = 3). O,P) Effect of O) MRPL12 knockdown or P) overexpression on ROS accumulation in HepG2 cells measured by DCFH‐DA probe (*n* = 3). Data are presented as mean ± SEM. t test, *P < 0.05, **P < 0.01, ***P < 0.001, ****P < 0.0001, and n.s., not significant.

To further explore the expression profile affected by lefamulin, RNA‐Seq analysis was performed in HepG2 cells treated by lefamulin (Figure S4A, Supporting Information). The expression of mRNAs was considered to be significantly different if the difference between Ctrl‐treated HepG2 cells versus Lefamulin‐treated HepG2 cells and Ctrl‐treated HepG2‐derived xenografts versus Lefamulin‐treated HepG2‐derived xenografts was >1.2‐fold at *P*‐adj value < 0.05. We got 3 downregulated genes both in lefamulin treated cells and tumors (**Figure** **4**A). We confirmed that lefamulin reduced the mRNA and protein levels of MRPL12 in cells and tumors (Figure 4B–F; Figure S4B, Supporting Information). However, lefamulin treatment showed no significant effect on SNHG19 and NTHL1 (Figure S4C, Supporting Information). MRPL10 and MRPL11 are two MRPs that are highly homologous to MRPL12 (Figure S4D, Supporting Information), but we didn't observe significant change in MRPL10 and MRPL11 expression upon lefamulin treatment (Figure S4E,F, Supporting Information). In addition, we found that MRPL12 expression was upregulated in sorafenib resistant cells and tumors (Figure S4G–J, Supporting Information). Thus, we speculated that lefamulin may affect mitochondrial function via regulation of MRPL12. To reveal the clinical relevance and functions of MRPL12 in HCC, we analyzed the MRPL12 expression in public TCGA transcriptome datasets. We found that MRPL12 expression was significantly upregulated and positively associated with worse overall survival in HCC patients (Figure 4G,H), and sorafenib‐treated liver cancer patients whose tumors expressed higher expression of MRPL12 were correlated with lower probability of overall survival (Figure 4I). Moreover, Gene Expression Omnibus (GEO) database analysis revealed that MRPL12 expression was upregulated in tumors of sorafenib non‐responsive HCC patients (Figure 4J), suggesting the potential of MRPL12 as a biomarker to predict the efficacy of sorafenib in HCC patients.

Next, to figure out whether MRPL12 is the key factor mediating‐sorafenib resistance, we manipulated the MRPL12 expression in the presence of sorafenib or lefamulin. We found that MRPL12 knockdown remarkably increased the sensitivity of HCC cells to sorafenib, while MRPL12 overexpression significantly reversed the sensitization effect of lefamulin as indicated by cell viability assays (Figure 4K–N), EDU incorporation and cell apoptosis assays (Figure S4K–N, Supporting Information). Moreover, MRPL12 knockdown augmented (Figure 4O), while enforced MRPL12 expression attenuated the ROS production induced by sorafenib or lefamulin combination treatment (Figure 4P). Collectively, lefamulin‐mediated MRPL12 downregulation and mitochondrial dysfunction mainly contribute to the vulnerability of HCC to sorafenib.

### 2.5 Lefamulin Targets ILF3 to Sensitize HCC to Sorafenib

**Figure 5:**
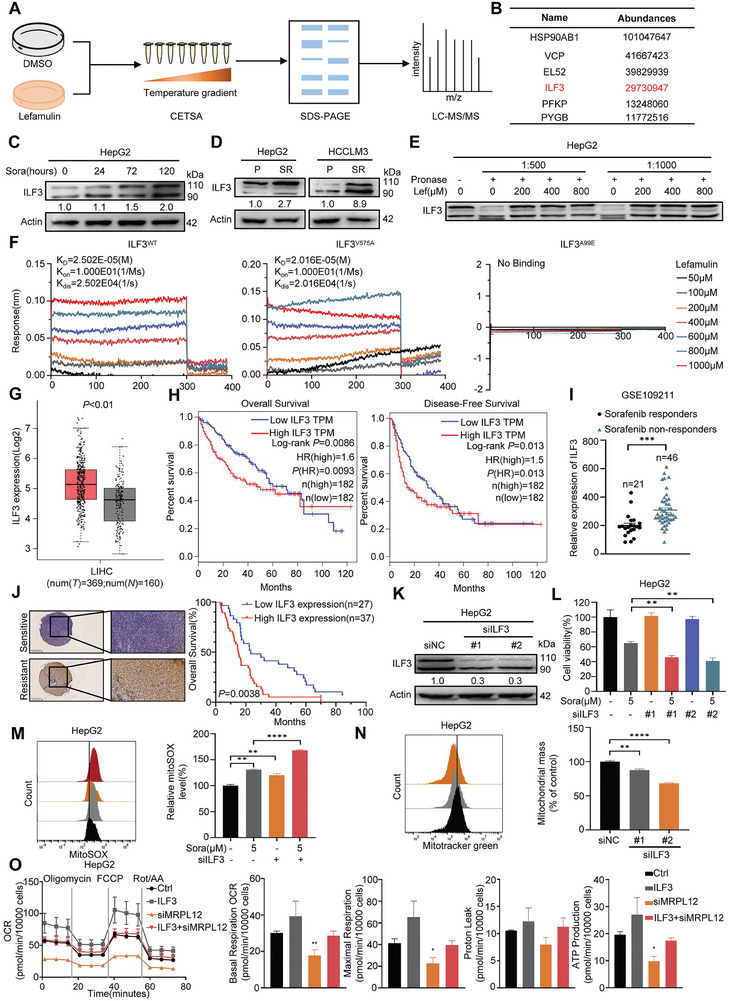
Lefamulin targets ILF3 to sensitize HCC to sorafenib. A) Schematic diagram of the CETSA method and mass spectrometry analysis. B) The 6 candidate proteins with high abundances identified by mass spectrometry analysis. C) Western blot was carried out to examine protein expression of ILF3 in HepG2 cells treated with 5 × 10^−6^ m sorafenib for 0, 24, 72, and 120 h. D) Western blot was carried out to examine protein expression of ILF3 in sorafenib‐sensitive (P) and resistant (SR) HepG2 and HCCLM3 cells. E) DARTS was used to evaluate the binding of lefamulin with ILF3. The expression of ILF3 was detected by western blot. F) BLI analysis showing the affinity of lefamulin for the ILF3^WT^, ILF3^V575A^ and ILF3^A99E^ protein. G) The expression of ILF3 in HCC (*n* = 369) and non‐tumor tissues (*n* = 160) (*P* < 0.01) from GEPIA database. Based on the selected TCGA tumors and TCGA normal + GTEx normal datasets performed difference analysis. H) Kaplan‐Meier overall survival (Log‐rank *P =* 0.0086) and disease‐free survival (Log‐rank *P =* 0.013) curves of HCC patients, using the TCGA datasets in the GEPIA database. High and low expression of ILF3 were stratified by the median. I) ILF3 mRNA levels in tumors between sorafenib responders (*n* = 21) and sorafenib non‐responders (*n* = 46) from GEO database. J) Left, representative images of IHC staining of ILF3 in sorafenib‐resistant or sorafenib‐sensitive liver cancer tissues. Scale bar, 500 µm (left), 100 µm (right). Right, Kaplan‐Meier analysis of liver cancer patients’ overall survival grouped by low or high expression of ILF3. *P*‐values were determined by Log‐rank test, *P =* 0.0038. K) Western blot was carried out to examine protein expression of ILF3 in HepG2 cells transfected with or without siILF3. L) Effect of ILF3 knockdown on sorafenib sensitivity in HepG2 cells. The cells were treated for 72 h. Cell viability was measured by CCK‐8 assay (*n* = 3). M) Effect of ILF3 knockdown and/or sorafenib treatment on ROS accumulation in HepG2 cells measured by DCFH‐DA probe (*n* = 3). *N*) Effect of ILF3 knockdown on mitochondrial mass in HepG2 cells measured by mitotracker green probe (*n* = 3). O) Oxygen consumption rate (OCR) analysis using Seahorse analysis revealed compromised mitochondrial function in HepG2 cells transfected with siMRPL12 and/or ILF3‐Flag. Basal respiration, ATP production, maximal respiration, and spare respiratory capacity were identified, respectively (*n* = 3). Data are presented as mean ± SEM. t test, *P < 0.05, **P < 0.01, ***P < 0.001, and ****P < 0.0001.

To find out the direct target of lefamulin, we carried out a cellular thermal shift assay (CETSA) followed by mass spectrometry analysis (**Figure** **5**A; Figure S5A, Supporting Information), and 27 proteins were identified as potential targets of lefamulin (Figure 5B; Table S2 and Data file S2, Supporting Information). Among the candidate proteins, ILF3 attracted our attention. We found that sorafenib long‐term stimulation promoted ILF3 expression (Figure 5C; Figure S5B, Supporting Information), and ILF3 expression was upregulated in sorafenib resistant cells and tumors (Figure 5D; Figure S5C, Supporting Information). Drug affinity responsive target stability (DARTS) and CETSA experiments verified the binding of lefamulin with ILF3 (Figure 5E; Figure S5D,E, Supporting Information). To further identify the binding site for lefamulin in ILF3, we conducted a molecular docking simulation to predict the interaction of lefamulin with the ILF3 protein using the solution NMR structure of DRBM2 domain of ILF3 from PDB (2L33, score = −7) and predicted crystal structure of the whole domain of ILF3 from AlphaFold (AF‐Q12906‐F1, score = −5.074). Results indicated that lefamulin may bind to the ILF3 protein with the residues Alanine‐99 (A99) or Valine‐575 (V575) (Figure S5F,G, Supporting Information). Accordingly, we generate two ILF3 mutants, ILF3^V575A^ and ILF3^A99E^, CETSA and DARTS results showed that the residue A99 site was crucial for the binding of lefamulin with ILF3 (Figure S5H,I, Supporting Information). Next, we expressed and purified the recombinant ILF3^WT^ protein, ILF3^V575A^ and ILF3^A99E^ mutant protein with a prokaryotic expression system (Figure S5J, Supporting Information). Bio‐layer interferometry (BLI) assay further confirmed that lefamulin interacted with ILF3^WT^ protein (*K*_D_ = 25.02 × 10^−6^ m) and ILF3^V575A^ mutant protein (*K*_D_ = 20.16 × 10^−6^ m) with high affinity, however, lefamulin could not bind to ILF3^A99E^ mutant protein (Figure 5F), which further verified the importance of ILF3 Ala‐99 residue for lefamulin binding.

To reveal the clinical relevance and functions of ILF3 in HCC, we analyzed the public TCGA transcriptome datasets and the GEO database. We found that ILF3 expression was significantly upregulated in HCC patients (Figure 5G), and high ILF3 expression was associated with poor overall survival and disease‐free survival (Figure 5H). GEO database analysis revealed that ILF3 expression was upregulated in tumors of sorafenib non‐responsive HCC patients (Figure 5I). To further validate our findings, we analyzed by ILF3 immunohistochemistry 64 HCCs from patients who underwent sorafenib treatment following surgery. Reduced immunostaining of ILF3 was associated with improved patient survival following treatment with sorafenib (*P* = 0.0038) (Figure 5J).

Next, to further confirm the role of ILF3 in sorafenib resistance, we knockdown ILF3 and detected the susceptibility of HCC cells to sorafenib (Figure 5K). As expected, ILF3 knockdown enhanced the sensitivity of HCC cells to sorafenib as indicated by cell viability and apoptosis assay (Figure 5L; Figure S5K, Supporting Information). Moreover, ILF3 knockdown also enhanced sorafenib‐mediated ROS production and reduced mitochondrial mass (Figure 5M,N), indicating that ILF3 may affect mitochondrial function. Given that lefamulin impaired mitochondrial function by downregulating MRPL12, we speculated that ILF3 may affect mitochondrial function via MRPL12. Indeed, ILF3 overexpression enhanced mitochondrial respiration and was reversed by MRPL12 knockdown in HepG2 cells, as indicated by basal OCR, ATP‐linked OCR, maximal respiration, and spare respiratory capacity (Figure 5O). To this end, we demonstrate that lefamulin increases the sensitivity of sorafenib in HCC by targeting ILF3, and ILF3 could serve as a biomarker to predict the efficacy of sorafenib in HCC patients.

### 2.6 ILF3 Is a Transcriptional Activator of MRPL12

**Figure 6:**
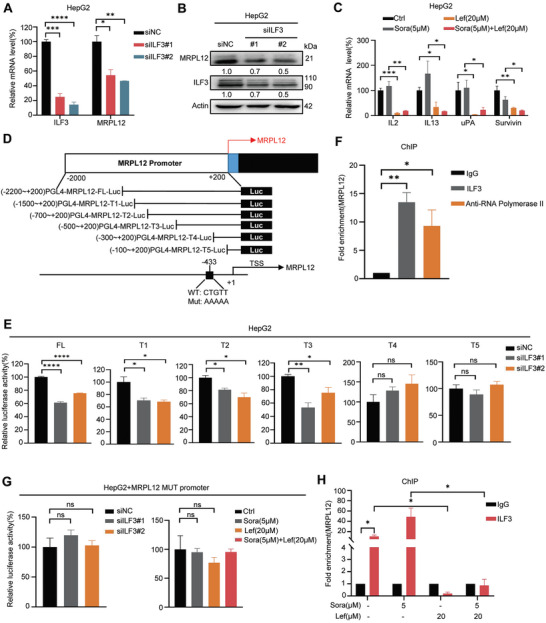
ILF3 is a transcriptional activator of MRPL12. A) The mRNA (*n* = 3) and B) protein expression of MRPL12 in HepG2 cells with or without ILF3 knockdown. C) The mRNA expression of ILF3 downstream target genes, including *IL2*, *IL13*, *uPA* and *Survivin* in HepG2 cells treated with lefamulin and/or sorafenib (*n* = 3). D) The construction of pGL4‐Luc reporter plasmids containing DNA fragments serially deleted from ‐2000 to +200 bp or mutant of the MRPL12 promoter. E) Fold change in luciferase activity driven by MRPL12‐promoter reporter under ILF3 siRNA or control siRNA transfection in HepG2 cells (*n* = 3). F) ChIP assays to determine the enrichment of ILF3 protein in the MRPL12 promoter in HepG2 cells (*n* = 3). G) Fold change in luciferase activity driven by MRPL12‐mutant promoter reporter under ILF3 siRNA or control siRNA treatment, or lefamulin and/or sorafenib treatment in HepG2 cells (*n* = 3). H) ChIP assays were used to determine the enrichment of ILF3 protein in the MRPL12 promoter under the treatment of lefamulin and/or sorafenib in HepG2 cells (*n* = 3). Data are presented as mean ± SEM. t test, *P < 0.05, **P < 0.01, ***P < 0.001, ****P < 0.0001, and n.s., not significant.

The above results showed that MRPL12 downregulation is the key factor for lefamulin to sensitize HCC to sorafenib, and ILF3 is the direct target of lefamulin, we wondered whether lefamulin reduced MRPL12 expression in an ILF3‐dependent manner. Firstly, we found that ILF3 knockdown reduced (**Figure** **6**A,B), while ILF3 overexpression enhanced MRPL12 mRNA and protein expressions (Figure S6A,B, Supporting Information). However, ILF3 knockdown did not change the expression of MRPL10 and MRPL11 (Figure S6C, Supporting Information). Additionally, lefamulin treatment reduced ILF3 downstream target gene expression, like IL2, IL13, uPA and Survivin (Figure 6C; Figure S6D, Supporting Information). Given that ILF3 functions as a transcription factor to activate or repress gene expression,^[^ ^22^, ^23^ ^]^ we speculated that ILF3 may transcriptionally regulate MRPL12 expression. Firstly, we constructed pGL4‐MRPL12‐Luc reporter plasmids with different regions of MRPL12 promoter (Figure 6D) and detected the promoter activities under the condition of ILF3 knockdown. According to the luciferase reporter assay results, we surmised that the binding site of ILF3 in MRPL12 promoter may localize between nucleotides −500 to +200 (Figure 6E). Chromatin immunoprecipitation (ChIP) assay further revealed a significant enrichment of ILF3 protein at the MRPL12 promoter in HepG2 cells (Figure 6F), suggesting the binding of ILF3 to MRPL12 promoter. Indeed, we found a consensus CTGTT sequence,^[^ ^24^, ^25^ ^]^ the putative ILF3 binding site in nucleotides −435 to −431 of MRPL12 promoter. Furthermore, we generated mutant constructs of the putative binding site and found no significant changes of luciferase activities when ILF3 knockdown (Figure 6G). Consistently, sorafenib treatment increased the luciferase activities of reporter constructs which include the ILF3 binding site, and was blocked by lefamulin (Figure S6E, Supporting Information). However, sorafenib and lefamulin showed no effect on the mutant luciferase constructs (Figure 6G). Meanwhile, we found that sorafenib treatment increased the enrichment of ILF3 at the MRPL12 promoter, and lefamulin significantly reduced the ILF3 enrichment (Figure 6H). Moreover, EMSA experiments conveyed that ILF3 protein formed a DNA‐protein complex shift with the WT‐MRPL12 probe, but not MUT‐MRPL12 probe (Figure S6F, Supporting Information), further demonstrating the direct binding of ILF3 protein to MRPL12 promoter. Together, these results provide convincing evidence that ILF3 is a transcriptional activator of MRPL12 expression and sorafenib triggered ILF3‐MRPL12 axis confers HCC cells drug resistance.

### 2.7 Lefamulin Inhibits GCN5/CBP‐Mediated Acetylation of ILF3 and Subsequent Transcriptional Activation

**Figure 7:**
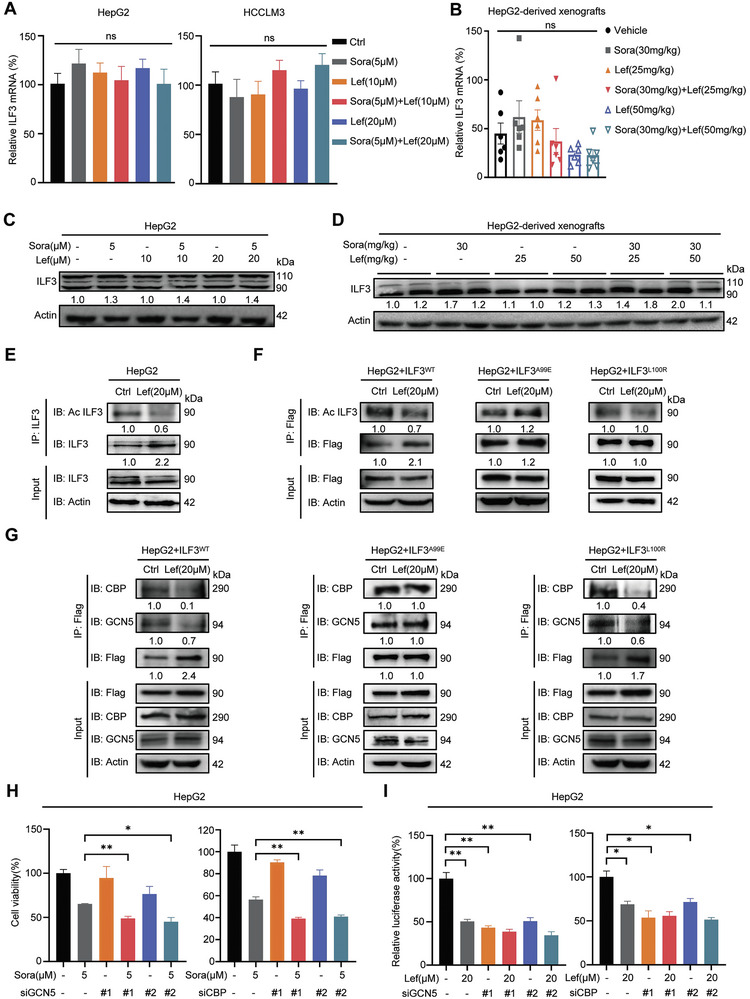
Lefamulin inhibits GCN5/CBP‐mediated acetylation of ILF3 and subsequent transcriptional activation. A,B) RT‐qPCR was carried out to examine mRNA expression of *ILF3* in A) HepG2, HCCLM3 cells (*n* = 3) and B) HepG2‐derived xenografts (*n* = 6) treated with lefamulin and/or sorafenib. C,D) Western blot was carried out to examine protein expression of ILF3 in C) HepG2 cells and D) HepG2‐derived xenografts treated with lefamulin and/or sorafenib. E,F) Western blot was carried out to examine the expression of E) acetylated ILF3 or F) acetylated Flag‐ILF3 in HepG2 cells in the presence or absence of 20 × 10^−6^ m lefamulin. G) Western blot was carried out to examine the interaction between ILF3 and GCN5 or CBP in HepG2 cells transfected with ILF3^WT^‐Flag, ILF3^A99E^‐Flag or ILF3^L100R^‐Flag in the presence or absence of 20 × 10^−6^ m lefamulin. H) Cell viability was examined in GCN5 or CBP‐knockdown HepG2 cells treated with 5 × 10^−6^ m sorafenib by CCK‐8 assay (*n* = 3). I) Luciferase activity was examined in GCN5 or CBP knockdown HepG2 cells treated with 20 × 10^−6^ m lefamulin by dual luciferase analysis kit (*n* = 3). Data are presented as mean ± SEM. t test, *P < 0.05 and **P < 0.01.

Since lefamulin targets ILF3 and inhibits following MRPL12 transcriptional expression, we next wondered how lefamulin regulates ILF3 function. Surprisingly, ILF3 gene and protein expressions showed no obvious change upon lefamulin treatment in HCC cells and tumors (**Figure** **7**A–D; Figure S7A,B, Supporting Information). In addition, lefamulin treatment did not affect the intracellular localization of ILF3 (Figure S7C, Supporting Information). Therefore, we speculated that lefamulin may affect the transcriptional activity of ILF3 by post‐translational modification. Different post‐translational modifications of ILF3 have been reported including phosphorylation,^[^ ^26^, ^27^ ^]^ demethylation^[^ ^28^ ^]^ and acetylation.^[^ ^29^ ^]^ Although sorafenib significantly increased the phosphorylation level of ILF3, we found lefamulin itself did not affect the ILF3 phosphorylation, indicating that lefamulin regulated ILF3 function in other ways (Figure S7D, Supporting Information). It was reported that ILF3 Lysine‐100 (L100) and Lysine‐105 (L105) acetylation initiated the transcription of downstream target genes.^[^ ^29^ ^]^ Previously, we have confirmed that lefamulin binds to Ala‐99 residue of ILF3 which is next to Lys‐100 spatially, we speculated that lefamulin may affect Lys‐100 acetylation of ILF3. Indeed, lefamulin significantly reduced the acetylation level of endogenous ILF3 (Figure 7E). We also generated Flag‐tagged ILF3^WT^, and ILF3^A99E^ (lost the binding ability of lefamulin), ILF3^L100R^ (mimic deacetylation) mutants. We found that lefamulin significantly reduced the acetylation level of ILF3^WT^, but did not change the acetylation level of ILF3^A99E^ or ILF3^L100R^ (Figure 7F). GCN5 and CBP are two acetyltransferases predicted to be responsible for ILF3 acetylation.^[^ ^29^ ^]^ We found that lefamulin disrupted the interaction between ILF3^WT^ or ILF3^L100R^ and GCN5/CBP, while showed no effect on the ILF3^A99E^ mutant (Figure 7G), suggesting that lefamulin inhibited GCN5/CBP‐mediated Lys‐100 acetylation of ILF3 by binding to Ala‐99. Meanwhile, we manipulated the GCN5 or CBP expression in the presence of sorafenib or lefamulin (Figure S7E,F, Supporting Information), and found that knockdown GCN5 or CBP promoted sorafenib‐induced cell death (Figure 7H) and reduced MRPL12 promoter activity (Figure 7I). Next, we employed the GCN5 inhibitor MB‐3 and CBP inhibitor PF‐CBP1 to further validate our findings. Indeed, MB‐3 and PF‐CBP1 downregulated the acetylation levels of ILF3 (Figure S7G, Supporting Information), promoted sorafenib‐induced cell death (Figure S7H, Supporting Information) and reduced MRPL12 promoter activity (Figure S7I, Supporting Information). Collectively, these data suggest that lefamulin disrupts GCN5/CBP‐mediated acetylation of ILF3, and subsequently inhibits the transcriptional activity of ILF3, enhancing the sensitivity of sorafenib in HCC.

### 2.8 Lefamulin Sensitized HCC Cells to Sorafenib and Regorafenib in an Immune‐Competent Mouse Model

**Figure 8:**
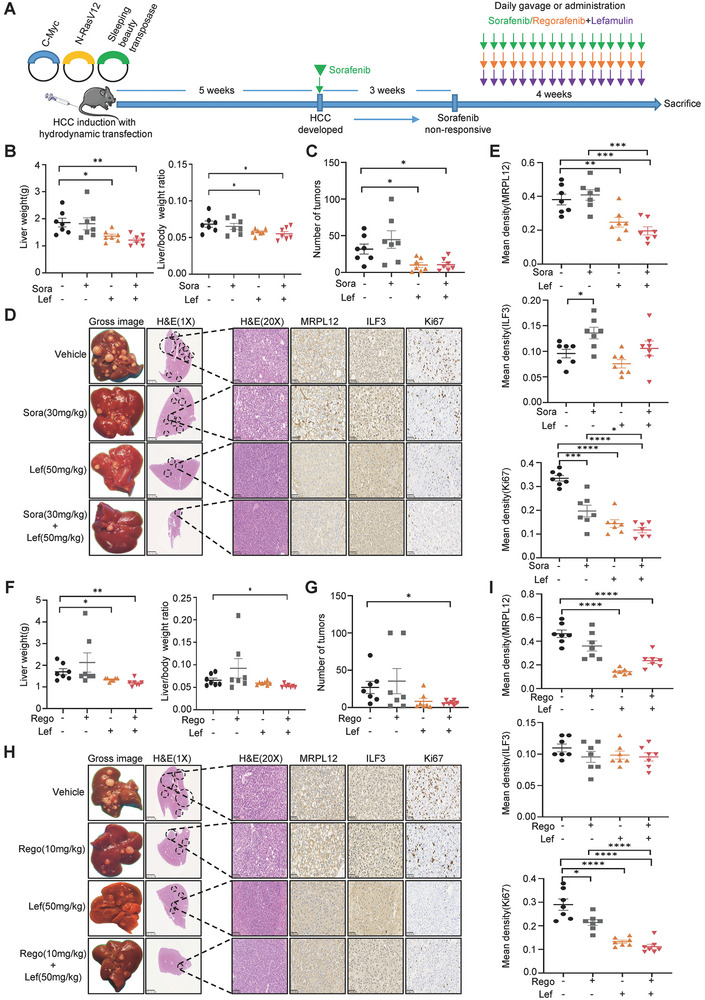
Lefamulin sensitized HCC cells to sorafenib and regorafenib in an immune‐competent mouse model. A) Schematic diagram of the treatment regimen with sorafenib/regorafenib, lefamulin alone or the combination of sorafenib/regorafenib with lefamulin, *n* = 7. B,F) Liver weight, liver/body weight ratio and C,G) number of tumors in hydrodynamic injection animal model were shown (*n* = 7). D,H) Representative gross images of HCC tumors, H&E staining (Scale bar, 2.5 mm (left), 100 µm (right)) and IHC images of MRPL12, ILF3 and Ki67 (Scale bar, 50 µm) in hydrodynamic injection animal model. E,I) Quantification of MRPL12, ILF3 and Ki67 expression (*n* = 7). Data are presented as mean ± SEM. t test, *P < 0.05, **P < 0.01, ***P < 0.001, and ****P < 0.0001.

In addition to HCC xenografts established in an immune‐deficient mouse background, we also investigated the potential of lefamulin alone or combined with sorafenib and regorafenib for liver cancer treatment in a genetically engineered immune‐competent mouse model of HCC. We performed hydrodynamic tail vein transfection of activation forms of C‐Myc and N‐RasV12 proto‐oncogenes, which are stably integrated into the genome of hepatocytes following transient expression of sleeping beauty transposase (SBT), for HCC induction (**Figure** **8**A). It was reported that 2 to 3 weeks post‐treatment of sorafenib after tumor development did not result in any survival advantage in the hydrodynamic transfection induced liver cancer model, which resembles the clinical situation of sorafenib nonresponsive patients.^[^ ^30^ ^]^ According to the pre‐experimental results, we found that HCC tumor started to develop after 5 weeks post‐injection. At this point, we first treated the HCC model with sorafenib alone for 3 weeks, and then sorafenib, regorafenib, lefamulin or their combinations was administered in this sorafenib non‐responsive tumor model. We found lefamulin alone or in combination with sorafenib (Figure 8B,C) or regorafenib (Figure 8F,G) led to significantly reduced tumor burden, as indicated by a reduction in liver weight, as well as the ratio of liver weight to body weight, and the number of tumor nodules. In addition, gross images, HE and IHC staining also revealed that lefamulin alone or in combination with sorafenib/regorafenib treatment could reduce tumor densities, and accompanied by a lower level of Ki67‐positive cells and MRPL12 expression, but did not affect ILF3 expression (Figure 8D,E,H,I). Taken together, these data clearly show the critical role of lefamulin in enhancing the efficacy of sorafenib and regorafenib treatment and reversing drug resistance in vivo, suggesting a new combination strategy for treatment of HCC.

## 3 Discussion

Sorafenib was the first multi‐kinase inhibitor approved by the US Food and Drug Administration (FDA) as a first‐line treatment for unresectable advanced HCC.^[^ ^31^ ^]^ However, the overall survival benefit of the patients is less than 3 months due to a high rate of drug resistance and tumor relapse.^[^ ^5^ ^]^ Similar low benefits are also reported in other multi‐kinase inhibitors, including regorafenib, lenvatinib and cabozantinib.^[^ ^32^, ^33^ ^]^ Therefore, further investigation of the molecular mechanisms underlying the therapeutic resistance and develop novel therapeutic drugs and combinational strategies are still urgently needed. Here, we identified an FDA‐approved antibiotic lefamulin that overcomes targeted therapy resistance in vitro and in vivo HCC models. Lefamulin targeting ILF3 exerts an antitumor effect in preclinical tumor models either alone or in combination with sorafenib or regorafenib, highlighting its potential in HCC treatment. ILF3 is a transcriptional activator of MRPL12, and lefamulin inhibits GCN5/CBP‐mediated acetylation of ILF3 and its transcription activity, leading to MRPL12 expression downregulation and mitochondrial dysfunction. Our study provides a proof‐of‐concept demonstration for a potential strategy to overcome targeted therapy resistance in HCC patients by targeting the ILF3‐MRPL12 axis.

Drug repurposing, the application of an existing therapeutic to a new disease indication, holds promise of rapid clinical impact at a lower cost than de novo drug development.^[^ ^34^ ^]^ In search of compounds that enhance sorafenib efficacy, we screened a library comprising 1430 FDA‐approved drugs for their ability to sensitize HCC cells to sorafenib. We found lefamulin alone or combined with sorafenib/regorafenib/lenvatinib significantly inhibited HCC growth in vitro and in vivo. Lefamulin is the first pleuromutilin antibiotic used for systemic treatment of bacterial infections in humans.^[^ ^7^ ^]^ Several anti‐infection drugs exist that have shown clinical benefit in randomized clinical trials and are supported by additional preclinical evidence.^[^ ^35^ ^]^ Thus, further study should test the combination of lefamulin and sorafenib/regorafenib in trials, especially in patients with advanced HCC.

Mitochondria is the “powerhouse” of cellular metabolic functions under pathophysiological conditions, and emerging data suggest that dysregulated mitochondrial biogenesis, ROS and redox homeostasis instigate drug resistance.^[^ ^36^, ^37^ ^]^ MRPL12 functions in maintaining the structural and functional integrity of mitoribosome, and plays an essential role in regulating mitochondrial respiration and energy homeostasis.^[^ ^38^ ^]^ However, the function and regulation of MRPL12 in HCC development and drug resistance are little known so far. We found that MRPL12 expression was significantly upregulated in sorafenib non‐responsive HCC patients and associated with poor prognosis. These observations highlight the significance of MRPL12 in the HCC progression and sorafenib resistance. It is reported that MRPL12 participates in the translation of mitochondrial proteins, activation of mtDNA transcription by directly binding to mitochondrial RNA polymerase, initiation of mitochondrial biosynthesis, and regulation of mitochondrial OXPHOS.^[^ ^39^ ^]^ Since lefamulin downregulates MRPL12 expression, and MRPL12 overexpression significantly reversed the sensitization effect of lefamulin, we speculated that lefamulin may affect mitochondrial function via MRPL12. Indeed, lefamulin decreased mitochondrial numbers and oxidative phosphorylation levels in HCC cells, while enforced MRPL12 expression attenuated lefamulin and sorafenib induced ROS production. Interestingly, we observed that sorafenib or lefamulin alone had slight effect on mitochondrial integrity, however, the combination treatment significantly decreased mitochondrial quality and function. We proposed that lefamulin‐mediated MRPL12 downregulation leads to unhealthy mitochondria which increased the sorafenib susceptibility. The combination of sorafenib and lefamulin can augment the effect of sorafenib or lefamulin, thus contributing to sharp mitochondrial number loss and mitochondrial damage, and subsequent cell death. Accumulating evidence has demonstrated that targeting mitochondria could enhance the efficiency of cancer therapy including TKIs, chemotherapy drugs and immune checkpoint inhibitors, which is attributed to the essential role of mitochondria in the regulation of cancer cell apoptosis, metabolism.^[^ ^40^, ^41^, ^42^, ^43^, ^44^ ^]^ Given that lefamulin exhibits a sensitizing effect by targeting the mitochondria, we speculated that lefamulin may also improve the therapeutic efficacy of targeted agents, cytotoxic drugs, and immune therapeutics. Our present study establishes an innovative connection between the metabolic alterations induced by MRPL12 and sorafenib resistance of HCC and also highlights the significance of lefamulin in reversing drug resistance.

Accumulating evidence has revealed that ILF3 is highly expressed in various cancers, and closely related to poor prognosis.^[^ ^45^ ^]^ ILF3 participates in diverse cellular functions, such as mRNA stabilization, translation regulation, viral replication, and circRNA biogenesis.^[^ ^17^ ^]^ ILF3 is also involved in the EGF‐ERK signaling and plays an important role in systemic serine metabolic reprogramming and colorectal cancer development.^[^ ^18^ ^]^ However, the functional role of ILF3 in HCC progression remains poorly clarified. Here, we provide the first evidence showing that ILF3 expression was significantly upregulated in sorafenib non‐responsive HCC patients and associated with poor prognosis. Gain‐ and loss‐of‐function assays also demonstrated that ILF3 contributes to the vulnerability of HCC to sorafenib. Strikingly, lefamulin treatment does not affect ILF3 mRNA and protein expressions. Previous studies have identified ILF3 could be diversely modified, including methylation,^[^ ^28^ ^]^ phosphorylation,^[^ ^26^, ^27^ ^]^ acetylation,^[^ ^29^, ^46^ ^]^ and ubiquitination.^[^ ^18^, ^47^ ^]^ Phosphorylation of ILF3 was reported to affect its function in mRNA stabilization, cellular or viral translation regulation.^[^ ^17^ ^]^ It was reported that the Lys‐100 and Lys‐105 residues of ILF3 could be acetylated in the presence of acetyltransferases GCN5 and CBP.^[^ ^29^ ^]^ Given that lefamulin binds with Ala‐99 residue next to Lys‐100 of ILF3 spatially, we speculated that lefamulin may affect ILF3 acetylation. Indeed, lefamulin disrupts the interaction of ILF3 with GCN5/CBP and decreases the acetylation of ILF3 in Lys‐100. ILF3 was reported to be capable of both transcription activation and repression factor.^[^ ^22^ ^]^ ILF3 is responsible for a positive effect on IL2 and SP‐10 transcription,^[^ ^48^, ^49^ ^]^ a negative effect on STAT1, and cholesterol homeostasis related genes transcription.^[^ ^23^, ^50^ ^]^ Given that the ILF3 protein enriched at the MRPL12 promoter, we provide the first evidence showing that the MRPL12 gene is a new downstream target gene of ILF3. Post‐translational modifications of RNA binding proteins including acetylation are also crucial for their conventional function including RNA binding ability, mRNA stabilization ability, etc.^[^ ^51^, ^52^, ^53^, ^54^ ^]^ Therefore, we speculated that lefamulin decreased acetylation of ILF3 may also affect other functions of ILF3 including conventional effect on RNA metabolism and mediate various signal pathways in HCC. Interestingly, we found that sorafenib treatment upregulated the luciferase activity of the MRPL12 promoter and increased the enrichment of ILF3 at the MRPL12 promoter. In addition, we found sorafenib treatment promoted the phosphorylation of ILF3 surprisingly. Given that phosphorylation of ILF3 could affect its nuclear localization,^[^ ^26^, ^27^, ^55^ ^]^ we speculated that sorafenib treatment may affect the nuclear localization of ILF3 via phosphorylation, thus promoting its binding with promoters of target genes, which will be explored in future research.

One notable finding from our preclinical study is that lefamulin showed prominent tumor inhibitory effects in the hydrodynamic‐injection HCC models which resemble the clinical situation of sorafenib non‐responsive patients. Our findings support further clinical evaluation of lefamulin targeting ILF3, in combination with sorafenib/regorafenib in HCC patients. This provides a new clue for optimizing HCC therapy, the measurement of ILF3 expression may be an effective approach to predict a patient's response to sorafenib treatment. Firstly, sorafenib and lefamulin combinational treatment may be suitable for those patients with high expression of ILF3. Extensively, patients with high expression of ILF3 may benefit from the combinational treatment of lefamulin with other TKIs, chemotherapy drugs or immune checkpoint inhibitors.

**Figure 9:**
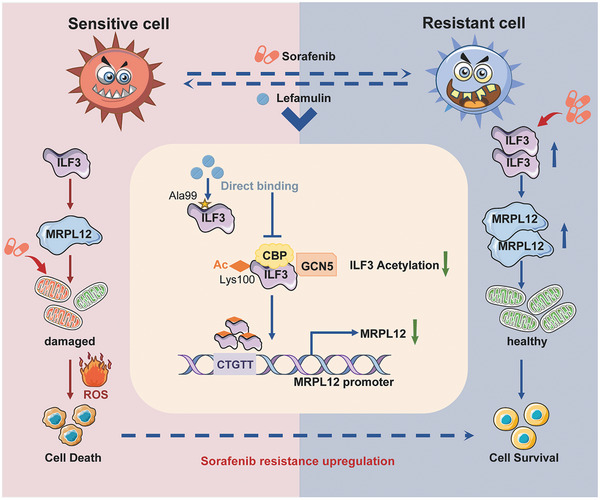
Schematic diagram summarizing how lefamulin overcomes sorafenib resistance via ILF3‐MRPL12 axis. Lefamulin directly targets Ala‐99 site of ILF3 and interferes with GCN5 and CBP‐mediated acetylation of Lys‐100, thus disrupting the ILF3‐mediated transcription of MRPL12 and subsequent mitochondrial biogenesis, mediating sorafenib resistance.

In summary, our findings have unveiled the critical role of the ILF3‐MRPL12 axis in the development of targeted therapy resistance in HCC, and uncovered a previously unrecognized role of an antibiotic, lefamulin, in reversing drug resistance. ILF3 or MRPL12 could serve as a prognostic biomarker and therapeutic target for HCC treatment. For the first time, we identified lefamulin targets ILF3 to overcome drug resistance through regulating mitochondrial homeostasis (**Figure** **9**). The therapeutic efficacy of lefamulin as a single agent or in combination with sorafenib/regorafenib supports its ongoing development or modification for further clinical translation. Given the poor response and high acquired drug resistance to sorafenib, our findings may also be of great value for future clinical development of ILF3 inhibitors in HCC and other malignancies.

## 4 Conclusion

Lefamulin overcomes drug resistance of HCC by targeting ILF3 and blocking GCN5 and CBP‐mediated acetylation of ILF3, which subsequently inhibits MRPL12 transcription and mediates mitochondrial homeostasis. Our study reveals that ILF3‐MRPL12 axis confers HCC cells drug resistance and ILF3 is a potential therapeutic target to overcome resistance, and lefamulin may be a novel combination therapy strategy for HCC treatment with TKIs.

## 5 Experimental Section

The details regarding the materials and methods, please refer to the Supporting Information.

## Conflict of Interest

The authors declare no conflict of interest.

## Author Contributions

H.Z. conceived the study and participated in the overall design, supervision, and coordination of the study. Y.Z. designed and performed most experiments and data analysis. S.‐T.Y. participated in drug screening experiments. S.‐T.Y. and S.‐Y.H. participated in the construction and identification of drug‐resistant cell lines. Y.C., Y.‐Q.Z., and Y.‐R.L. participated in molecular and cellular biological experiments. M.‐M.H., E.‐Y.W., and J.‐X.C. participated in animal studies. H.Z. and Y.Z. wrote the paper. L.‐Y.K. supervised the overall project. All authors read and approved the manuscript.

## Supporting information

Supporting Information

Supporting Data 1

Supporting Data 2

Supporting Data 3

## Data Availability

The data that support the findings of this study are available in the Supporting Information of this article.
