# Supplementary material for: Lefamulin Overcomes Acquired Drug Resistance via Regulating Mitochondrial Homeostasis by Targeting ILF3 in Hepatocellular Carcinoma
Source: Adv Sci (Weinh). 2024 Jun 14;11(30):2401789. doi: 10.1002/advs.202401789 (PMC11321631; doi:10.1002/advs.202401789)
Supplement: Supplementary file 1 — Supporting Information [file ADVS-11-2401789-s001.pdf]

ADVANCED SCIENCE
Open Access

Supporting Information

for *Adv. Sci.*, DOI 10.1002/advs.202401789

Lefamulin Overcomes Acquired Drug Resistance via Regulating Mitochondrial Homeostasis by Targeting ILF3 in Hepatocellular Carcinoma

Ying Zheng, Shengtao Ye, Shiyu Huang, Yang Cheng, Yanqiu Zhang, Yingrong Leng, Mengmeng He, Enyi Wu, Junxin Chen, Lingyi Kong and Hao Zhang**

WILEY-VCH

Supporting Information

Lefamulin Overcomes Acquired Drug Resistance via Regulating Mitochondrial Homeostasis by Targeting ILF3 in Hepatocellular Carcinoma

Ying Zheng, Shengtao Ye, Shiyu Huang, Yang Cheng, Yanqiu Zhang, Yingrong Leng, Mengmeng He, Enyi Wu, Junxin Chen, Lingyi Kong, and Hao Zhang**

The file includes:

Materials and Methods

Figures S1-S7

Tables S1-S4

Legend for Data file S1-S3

Other Supplementary Material for this manuscript includes the following:

Data file S1-S3

Supplementary materials and Methods

Animals

All animal care and experimental procedures were approved by the University Committee on Use and Care of Animals of the China Pharmaceutical University (Nanjing, China) (Resolution Number 2022-03-024). 4-week-old male Balb/c nude mice and C57BL/6J mice used for this study (16-20 g, specific pathogen-free class) were purchased from Charles River Laboratories Co., Ltd (Beijing, China).

For the subcutaneous tumor model, 4×10^6 HepG2 cells suspended in 100 μL serum-free DMEM and Matrigel (1:1) were subcutaneously injected into the right flank of Balb/c nude mice. When the xenografts were palpable, measuring the tumor size every 2 or 3 days using a digital caliper, calculating the tumor volumes with the formula: tumor volume = length $\times$ width^2 $\times$ 0.5. When tumor volume reached approximately 100 mm^3, the mice were randomized into treatment groups, including vehicle control, sorafenib (30 mg/kg/day, orally), lefamulin(25 or 50 mg/kg/day, intraperitoneally) or the combination, 6 mice per groups. Blood samples were collected before sacrificing the mice, then all the tumors were removed, weighed, and frozen in liquid nitrogen for further studies.

To generate hydrodynamic injection liver cancer mouse model, C57BL/6J mice were used and the procedure was performed as described previously. In brief, 2 μg plasmids encoding human C-Myc, 40 μg plasmids encoding human N-RasV12, along with 4 μg sleeping beauty transposase (SBT) , were diluted in 2 mL saline, filtered through 0.22 μm filter and injected into mouse livers via the tail vein in 5 to 7 seconds. Five weeks later, drug administration with sorafenib was initiated to mimic clinical sorafenib resistance process of advanced HCC patients.

3 weeks later, the mice were randomized into different treatment groups, including vehicle control, sorafenib (30 mg/kg/day, orally) or regorafenib (10 mg/kg/day, orally), lefamulin(50 mg/kg/day, intraperitoneally) or the combination, 7 mice per groups. After completing the therapy, tumor-bearing mice were sacrificed, and livers were harvested for further analysis.

Cell lines and cell culture

In this study, human HCC cell lines (HepG2, HCCLM3, Huh7, SNU449, MHCC-97H, SK-Hep-1, Bel7402 and Hep3B) and normal cell lines (L02, WRL68 and HEK293T) were purchased from Shanghai Cell Bank of the Chinese Academy of Sciences. These cells were cultured in the 10% fetal bovine serum (FBS; Gibco) basal medium supplemented with 1% penicillin-streptomycin (NCM) at 37 ℃ in the presence of 5% CO_2 in an incubator and routinely detected for mycoplasma.

Establishment and culture of sorafenib-resistant cells in vitro and in vivo

The isogenic resistant cell lines resistant to sorafenib (HepG2 SR and HCCLM3 SR) in vitro were established by exposing cells to escalating doses of sorafenib for approximately 6 months and maintained with low dose of sorafenib, and authenticated.

The sorafenib-resistant in vivo tumor cells were isolated from xenografts of sorafenib-resistant HCC mouse. A subcutaneous mouse model was constructed firstly, as follows described. In total, 4×10^6 HepG2 cells were implanted into the flank of Balb/c nude mice. After 4 weeks, mice with similar tumor burdens were subjected to receiving 30 mg/kg/day sorafenib (MCE) or vehicle.

After 8 weeks of treatment, mice were sacrificed, and tumor samples were cut into small pieces to isolate tumor cells for further investigation. The isolation process includes the following steps: subcutaneous HepG2-derived xenografts were excised and mechanically dissociated by gentle pipetting, digested with collagenase (Thermo Fisher Scientific), filtered through 70 μm cell strainer (BD Bioscience), and centrifuged at 1500 *g* for 5 min, cell sediments were exposed to red cell lysis buffer (Beyotime) for 10 min to remove the red blood cells, tumor cells were washed with D-Hanks (Solarbio) 3 times and resuspended in Dulbecco's modified eagle's medium (DMEM; Hyclone) supplemented with 10% FBS.

Cell growth and viability assay

Cell growth curves and cell viability were determined using the Cell Counting Kit-8 (CCK-8; MCE) assay according to the manufacturer's instructions.

For cell growth curves analysis, cells were seeded at a density of 3000 cells per well in 96-well plates and treated with different concentrations of lefamulin (MCE), and/or sorafenib, regorafenib and lenvatinib (MCE) for 24, 48, 72 and 96 hours. For cell viability assay, cells were seeded at a density of 8000 cells per well in 96-well plates and treated with different concentrations of lefamulin, and/or sorafenib and regorafenib for 48 hours. The absorbance was measured at 450 nm after incubation with 10 μL of CCK-8 reagent for 3 hours using Spectra-Max Plus 384 (Molecular Devices).

Combination effect analysis

Combinational treatments will result in synergistic, additive or antagonistic effects. The combination index (CI) values were calculated using CompuSyn software according to the Chou-Talalay method: CI < 1, synergistic; CI = 1, additive; CI > 1, antagonistic.

Colony formation assay

Cells were seeded at a density of 1×10^4 cells per well in 6-well plates and treated with different concentrations of lefamulin, and/or sorafenib, regorafenib and lenvatinib for 24 hours. The cells were allowed to grow in complete media for another 14 days. The colonies were washed with PBS and fixed in 4% paraformaldehyde (Servicebio) for 15 minutes at room temperature and then stained with crystal violet (Beyotime). Finally, the colony numbers (>50 cells) were counted.

EDU incorporation assay

Cells were seeded at a density of 8000 cells per well in 96-well plates and treated with different concentrations of lefamulin, and/or sorafenib, regorafenib and lenvatinib for 24 hours. The cell proliferation was determined by 5-ethynyl-2'-deoxyuridine (EDU) incorporation assay using BeyoClick™ EdU-488 imaging detection kit (Beyotime) according to the manufacturer's instructions.

Cell apoptosis assays

Cell apoptosis was determined by flow cytometry using Annexin V-Fluorescein isothiocyanate (FITC)/PI Apoptosis Detection Kit (Beyotime) according to manufacturer's instructions. In brief,

3×10^5 cells were seeded in 6-well plates and treated with different concentrations of lefamulin, and/or sorafenib for 48 hours. Then the cells were double stained with FITC-conjugated Annexin V and PI for 15 minutes at room temperature in the dark and analyzed by flow cytometer (BD).

RNA extraction, reverse transcription and RT-qPCR assay

Total RNA from cells or tumors was extracted using an RNA extraction kit (ES Science) according to the manufacturer's instructions. The concentrations were measured by measuring absorbance at 260 and 280 nm with NanoDrop (Thermo Fisher Scientific), and 1 μg of total RNA was subjected to synthesize cDNA using HiScript®II Q Select RT SuperMix (Vazyme). RT-qPCR was conducted in biological triplicates using SYBR Green reagent (Vazyme) on a LightCycler 480 II system (Roche). Expression levels were normalized to the expression of GAPDH. PCR primer sequences are listed in Table S3. A melting curve of each amplicon was determined to verify its specificity.

Co-immunoprecipitation (Co-IP) and western blot analysis

Cells and tumor samples were lysed by RIPA lysis buffer (YEASEN) with 1% PMSF (Beyotime) to extract the total proteins by centrifugation at 12,000 *g* for 10 minutes at 4 °C, and the protein concentrations were determined by BCA kit (Beyotime).

For Co-IP, 1 mg proteins were incubated with the appropriate antibodies overnight at 4 °C and then the complexes were incubated with Protein A/G Plus Agarose (Santa Cruz Biotechnology) for 4 hours at 4 °C, immune-complexes were washed five times with PBS, suspended in 2 × SDS

loading buffer and boiled for 10 minutes. The immune-complexes were subjected to subsequent western blot analysis.

For western blot analysis, equal amounts of immune-complexes or lysates were subjected to 8 to 12% SDS-PAGE gel separation and transferred to PVDF membranes (Bio-Rad). Membranes were blocked with 5% skim milk (Beyotime) in PBST buffer (PBS containing 0.2% Tween 20) for 2 hours at room temperature and immunoblotted with diluted primary antibodies overnight at 4 °C. The specific antibodies used in this study are listed in Table S4. After incubation with peroxidase-conjugated secondary antibodies (YEASEN) for 2 hours at room temperature, the immunoblots were subjected to electrochemiluminescence using an ECL kit (Vazyme) according to the manufacturer's instructions with a ChemiDoc XRS^+ imaging system (Bio-Rad), and quantified using Image Lab software. Actin was served as an internal control.

siRNA, plasmids, transfection, and site-directed mutagenesis

The plasmids expressing human ILF3 and MRPL12, as well as the different length of MRPL12 promoter plasmids, were constructed by standard subcloning separately. The small interfere RNA (siRNA) specifically targeting human ILF3, MRPL12, GCN5 or CBP were purchased from Ribobio. The primers used for plasmid construction and the sequences of siRNA are listed in Table S3. Cells were transfected with siRNA or plasmids using Lipofectamine 3000 (Invitrogen) in Opti-MEM (Invitrogen) according to the manufacturer’s instructions. After approximately 10 to 12 hours of incubation, the medium was changed to complete medium, gene knockdown or overexpression were detected by western blot or RT-qPCR analysis after transfection for 48 hours.

Cells transfected with pcDNA or siNC duplexes were used as control.

Measurement of ROS and superoxide in the mitochondria

Cells were seeded in 6-well plates (3 × 10^5 cells per well) and treated with drugs for 48 hours. Cells were harvested and washed with PBS, then incubated with 10 mM DCFH-DA (Beyotime) or MitoSOX Red (YEASEN) for 20 min at 37 °C without lighting. Celluar ROS or mitochondrial ROS was determined by flow cytometry. Experimental data were analyzed using FlowJo software.

Transmission electron microscopy

HepG2 cells were harvested after treatment and fixed with 2.5% glutaraldehyde fixative buffer (Beyotime). Then, cells were subjected to postfixation, dehydration and embedding to obtain the ultrathin sections. Sections were stained with 1% uranyl acetate and/or lead citrate. The morphology of mitochondria was acquired on a transmission electron microscope.

Mitochondrial mass determination

The mitochondrial mass was analyzed by MitoTracker Green staining (Beyotime). Cells were seeded in 6-well plates overnight and subjected to different treatments. MitoTracker Green was then incubated with cells for 15 minutes at 37 °C according to the manufacturer's instructions, and subjected to flow cytometric analysis.

Mitochondrial DNA quantification

Total DNA was extracted from cells using Genomic DNA Mini Preparation Kit with Spin Column (Vazyme). Relative mitochondrial DNA (mtDNA) and nuclear DNA (nDNA) were determined by RT-qPCR with D-Loop2 gene to represent mtDNA and G6PC gene to represent nDNA. The mitochondrial DNA copy numbers were indicated by the ratio of mtDNA to nDNA.

Immunofluorescence staining

For mitochondrial morphological observation, L02 and WRL68 cells were plated on glass coverslips in a dish and treated with different agents, then the cells were washed with PBS buffer and stained with MitoTracker @ Deep Red FM (Thermo Fisher Scientific) to examine the mitochondrial morphology for 40 min at 37 °C， fixed with 4% paraformaldehyde for 15 minutes.

For determination of intracellular distribution of ILF3, HepG2 and HCCLM3 cells were plated on glass coverslips in a dish and treated with different agents, fixed with 4% paraformaldehyde for 15 minutes.

The fixed cells were then permeabilized with 0.5% Triton X-100 for 30 min, followed by a blocking step in 1% bovine serum albumin for 2 hours at room temperature. And incubated with primary antibodies at 4 °C overnight, followed by incubated with fluorochrome-labelled secondary antibody for 2 hours at room temperature (YEASEN). Next, the cells were then washed with PBS 3 times, and the nuclei were counterstained with DAPI (Beyotime) for 5 min at room temperature. Images were captured using an ImageXpress Micro Confocal Platform (Molecular Devices).

Oxygen consumption rate detection

The oxygen consumption rate of cells was analyzed by a Seahorse XF96 Extracellular Flux Analyzer (Seahorse Biosciences) to monitor mitochondrial respiration in real time using Seahorse XF Cell Mito Stress Test Kit (Seahorse Biosciences) as described. In brief, 1×10^4 cells in 3 replicates were seeded into each well, and treated by different drugs or transfected with siRNA or plasmids. OCR was measured before and after the injection of 1.5 μM oligomycin, 1.0 μM FCCP, and 0.5 μM rotenone/antimycin A.

Cellular thermal shift assay (CETSA)

Briefly, HepG2 cells or HEK293T cells transfected with Flag-ILF3 was seeded in 10 cm culture dishes and treated with lefamulin or DMSO for 6 hours. Then cells were harvested and washed with PBS, then resuspended to a density of 5×10^6 cells/mL in PBS containing protease inhibitor and dispensed into 8 PCR tubes (100 μL/tube) and heated at 46 to 60 °C by a thermal cycler (eppendorf) for 3 minutes. Subsequently, the cells were lysed by freeze-thaw in liquid nitrogen, cell lysates were obtained by centrifugation at 15,000 *g* for 15 minutes at 4 °C and analyzed by western blot.

Drug affinity responsive target stability (DARTS)

Briefly, HepG2 cells or HEK293T cells transfected with Flag-ILF3 was seeded in 10 cm culture dishes and collected. The total protein was extracted using NP40 lysis buffer (Sigma-Aldrich), cell lysates were centrifuged at 18,000 *g* for 10 minutes at 4 °C. The supernatants from

independent biological replicates were aliquoted in equivalent volumes containing 100 μg of protein and incubated for 1 hour at 37 °C with or without lefamulin. Pronase was added simultaneously into all samples and incubated at 37 °C for 30 minutes, then protease inhibitor cocktail was added to stop reactions and SDS-PAGE was carried out to analyze the protein expression.

Expression and purification of the ILF3 protein

Firstly, we constructed the pET-28a plasmids expressing His-tagged wild-type or mutant ILF3 by transforming into Escherichia coli BL21 Star (DE3) cells. Then, ILF3 protein expression was induced by 0.5 mM isopropyl β-D-thiogalactopyranoside (IPTG) (Sangon Biotech) and incubated for 20 hours at 16 °C with shaking at 200 rpm. Next, bacterial cells were collected and washed with double distilled water, lysed in non-denatured lysis buffer by sonication, and collected the supernatant by centrifugation at 15,000 *g* for 30 minutes at 4 °C, the supernatant was filtered through 70 μm strainer and loaded onto a His-tag Purification Resin (Beyotime) overnight at 4 ℃ shaking slowly. Finally, the His-tagged ILF3 fusion protein was eluted with 50 mM imidazole (Sangon Biotech) analyzed by SDS-PAGE.

Bio-layer interferometry (BLI) analysis

The dose-dependent binding affinities between lefamulin and ILF3 was assessed by BLI with Octet RED96 (ForteBio). Lefamulin was prepared at different concentrations in kinetic buffer [PBS, 0.05% bovine serum albumin, 0.01% Tween 20]. WT ILF3 or mutant ILF3 was the ligand

and coupled to the equilibrated Ni-NTA biosensor tips (ForteBio, Menlo Park, CA), sensors that incubated in buffer without proteins background were binding controls. Assays were performed according to a standard protocol with a total volume of 200 μL per well in 96-well black plates at 30 ℃ and datas were analyzed by Octet data analysis software using a double reference subtraction method.

RNA-Sequencing (RNA-seq) analysis

The total RNA was extracted using TRIzol (Vazyme) reagent and reverse transcribed into cDNA from HepG2 cells treated with DMSO or lefamulin for 48 hours, and HepG2-derived xenografts treated with vehicle, sorafenib, lefamulin, or the combination. Then RNA-seq of cells and tumors were accomplished with the assistance of Shanghai Majorbio Biotechnology Co., Ltd. (Shanghai, China) and Beijing Novogene Co., Ltd. (Beijing, China), respectively. A fold change > 1.2 and *P*-adj value < 0.05 were defined as differential expression to screen downregulated genes using DESeq2 software to perform DEG analysis and enrichment analysis for further study. Then we conducted KEGG pathways and GO enrichment analysis. The transcriptome sequencing data have been deposited in NCBI Gene Expression Omnibus (GEO) under the following accession number: GSE252987 and GSE252988 .

Mass spectrum analysis

We identified the different band between DMSO and lefamulin treated cell lysates under the assistance of CETSA and SDS-PAGE. Protein reduction alkylation, enzymatic hydrolysis,

followed by peptide desalt, and mass spectrometry detection were conducted in turn. The mass spectrum analysis was accomplished with the assistance of Beijing Novogene Co., Ltd. (Beijing, China).

Chromatin immunoprecipitation (ChIP) assay

Chromatin immunoprecipitation (ChIP) assays were performed by EpiQuik TM Chromatin Immunoprecipitation Kit (Epigentek) according to the manufacturer's instructions. Briefly, 2×10^6 HepG2 cells were harvested after 48 hours treatment of DMSO, sorafenib, lefamulin, or the combination and crosslinked with 10 mL of PBS containing 1% formaldehyde. Sheared chromatin fragments (200 to 600bp) was immunoprecipitated with 3 μg of the anti-IgG, anti-RNA Polymerase II, or anti-ILF3 antibody for 1.5 hours at room temperature. Immunoprecipitated DNA and input DNA were analyzed by RT-qPCR to examine the occupancy of ILF3 in the promoters of MRPL12.

EMSA

The purified His-ILF3 protein and 25bp WT- or MUT-MRPL12-5'FAM probe (Tsingke Biotechnology) were used for EMSA assays. Approximately 10 μM of probe and 50 μM protein were used per reaction, reactions were carried out in a 10 μL volume at 20 °C for 30 min. The reaction products were loaded on a 8% EMSA gel and run at 4 °C and 80 V for 2 hours. DNA bands were visualized using UV light at 260 nm. The sequences of the probes used to show ILF3-MRPL12 binding were WT-MRPL12 promoter oligonucleotide

(5'-GAGGCTCTGCCTGTTCTGATCTGAA-3') and MUT-MRPL12 promoter oligonucleotide (5'-GAGGCTCTGCAAAAACTGATCTGAA-3') which located at -445 to -421 of MRPL12 promoter.

Luciferase reporter assay

The different promoter regions of MRPL12 were ligated into the pGL4 luciferase reporter vector, then several pGL4-MRPL12-Luc reporter plasmids were constructed to further explore the binding site of ILF3 on the promoters of MRPL12. HepG2 cells were grown in 24-well plates and co-transfected with luciferase reporter plasmids and a renilla reporter plasmid and then conducted to different treatments for 48 hours. Cells were lysed and subjected to luciferase reporter assay to examine firefly and renilla luciferase signals (considered as a negative control) using Dual Luciferase® Reporter Assay System (Vazyme) by SpectraMax Paradigm (Molecular Devices).

Plasma biochemistry

Blood samples supplemented with anticoagulant from animals were centrifugated at 4000 rpm for 10 minutes at 4 °C to obtain plasma. Plasma alanine aminotransferase (ALT), aspartate aminotransferase (AST), creatinine (CRE) and urea nitrogen (BUN) levels were measured in animals using standard enzymic procedures to evaluate liver or kidney injury according to the manufacturer's instructions (Nanjing Jiancheng Bioengineering Institute).

Histology and IHC analysis

Tumors and livers were fixed with 4% paraformaldehyde overnight, embedded in paraffin, and then cut into 5 μm sections used for H&E staining and IHC staining with specific antibodies against ILF3, MRPL12 and Ki67. Histological sections were scanned with a light microscope (Olympus) and the percentage of stained positive areas were quantified using Image J. At least six randomly chosen fields from each section were analyzed.

Human HCC samples

The human HCC tissue microarrays (LVC1609), consisting of 64 pairs of HCC tissues with comprehensive clinicopathological and follow-up data, were purchased from Shanghai WEIAOBIO Biotech. Tissue microarrays were stained with anti-ILF3 antibodies and scanned with a light microscope. Kaplan-Meier survival curves were generated and analyzed using the Log-rank test.

Public database analysis

The expression of MRPL12 or ILF3 in HCC and non-tumor tissues was investigated in GEPIA database (http://gepia.cancer-pku.cn/).

The Kaplan-Meier analysis was performed using HCC patients data retrieved from using the TCGA datasets in the GEPIA database and Kaplan-Meier plotter database (http://kmplot.com/analysis/) to investigate the prognostic significance of MRPL12 or ILF3 in HCC.

GEO2R analysis was conducted on the GSE109211 dataset

(http://www.ncbi.nlm.nih.gov/geo/geo2r/) to investigate the differential expression of MRPL12 or ILF3 between HCC patients who respond to sorafenib treatment and those who exhibited resistance.

Statistical analysis

All statistics are shown as means ± standard error of mean (SEM) at least 3 independent experiments of biological replicates and analyzed by GraphPad Prism 8.0 using analysis of variance (ANOVA) or two-tailed Student's *t* test. Survival curves were analyzed by Kaplan-Meier Log-rank (Mantel-Cox) *t* test. *$P < 0.05$, **$P < 0.01$, ***$P < 0.001$, ****$P < 0.0001$, and n.s., not significant.

Supplementary Figures

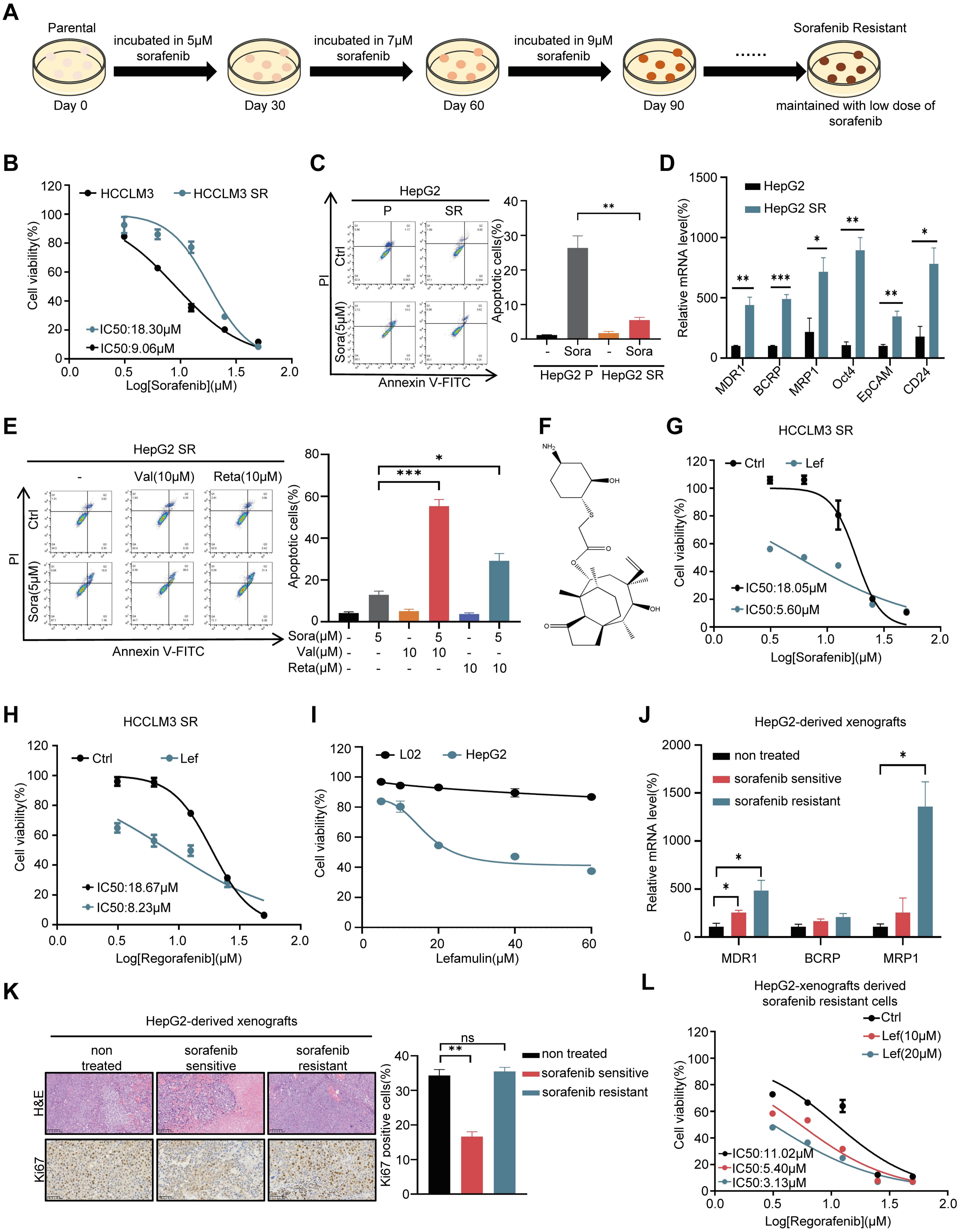

Figure S1. Identification of pleuromutilin class of antibiotics as sensitizers for sorafenib from FDA-approved library.

A) Schematic diagram of establishment of sorafenib-resistant HCC cells in vitro. **B**) The viability of sorafenib-resistant HCCLM3 SR cells and its corresponding parental cells HCCLM3 cells were analyzed by CCK-8 assay. Cells were treated with various concentrations of sorafenib for 48 hours (n = 3). **C**) Representative flow cytometric analysis of Annexin V-PI staining and quantification of apoptosis in sorafenib-sensitive and sorafenib-resistant HepG2 cells treated with 5 μM sorafenib for 48 hours (n = 3). **D**) RT-qPCR was carried out to examine mRNA expression of *MDR1*, *BCRP*, *MRP1*, *Oct4*, *EpCAM* and *CD24* in sorafenib-sensitive and sorafenib-resistant HepG2 cells (n = 3). **E**) Representative flow cytometric analysis of Annexin V-PI staining and quantification of apoptosis in HepG2 SR cells treated with 5 μM sorafenib and/or 10 μM valnemulin or retapamulin for 48 hours (n = 3). **F**) Chemical structure of lefamulin. **G**,**H**) HCCLM3 SR cells were treated with various concentrations of sorafenib (G) or regorafenib (H) alone, or in combination with 10 μM lefamulin for 48 hours, and cell viability was evaluated by CCK-8 assay (n = 3). **I**) Cell viability was analyzed in L02 and HepG2 cells treated with various concentrations of lefamulin (n = 3). **J**) RT-qPCR was carried out to examine mRNA expression of MDR1, BCRP and MRP1 in non-treated, sorafenib-sensitive and sorafenib-resistant tumor cells from HepG2-derived xenografts (n = 3). **K**) Representative images of hematoxylin and eosin (H&E) staining (Scale bar, 100 μm) for pathological analysis and IHC staining of Ki67 (Scale bar, 50 μm) for in situ analysis of cell proliferation of tumors applied to isolate cells (n = 3). **L**) HepG2-xenografts derived sorafenib resistant cells were treated with various

concentrations of regorafenib alone, or in combination with 10 or 20 μM lefamulin for 48 hours, and cell viability was evaluated by CCK-8 assay (n = 3). Data are presented as mean ± SEM. t test, *P < 0.05, **P < 0.01, ***P < 0.001, and n.s., not significant.

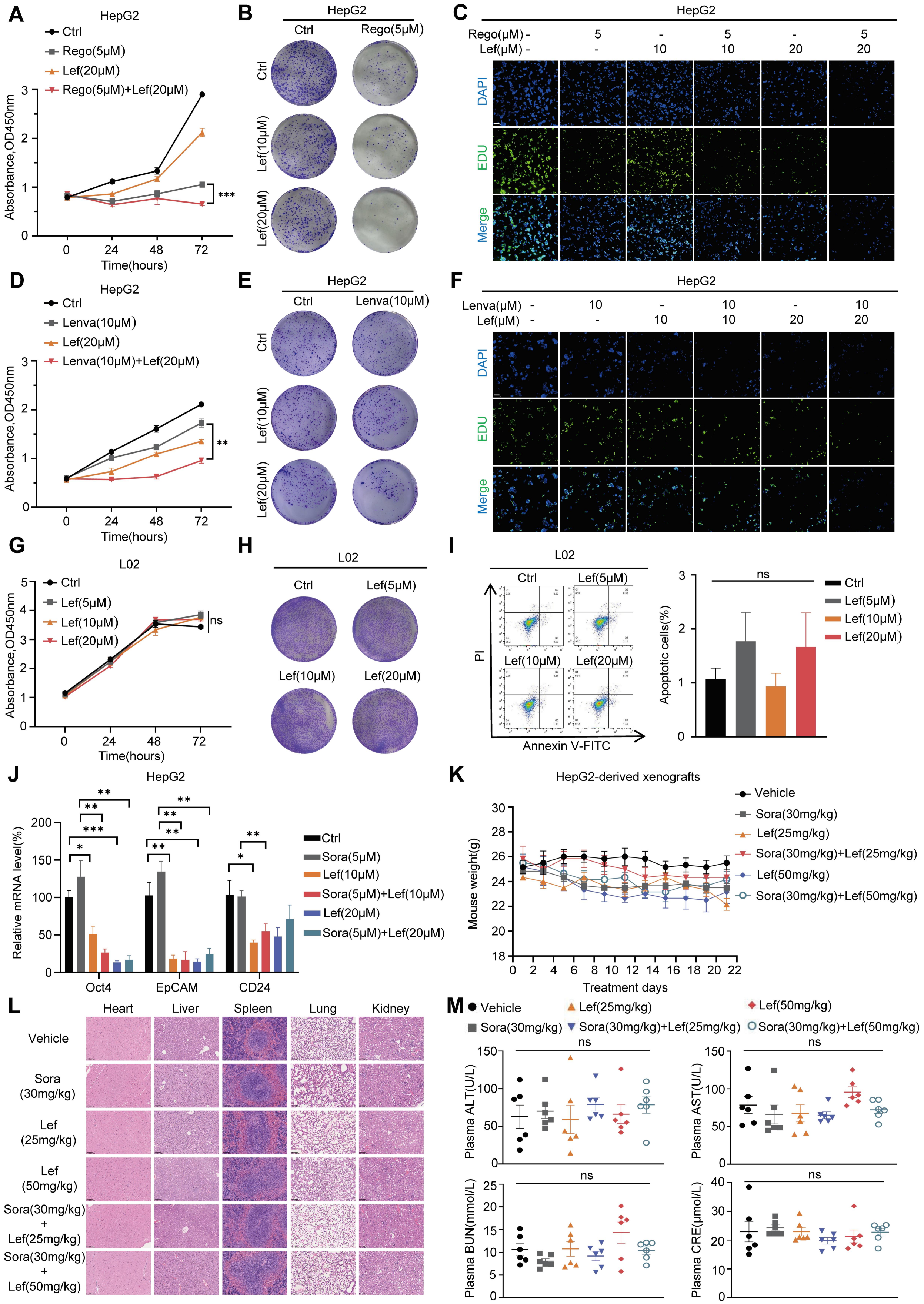
A
HepG2
Ctrl
Rego(5μM)
Lef(20μM)
Rego(5μM)+Lef(20μM)
Absorbance,OD450nm
Time(hours)
***
B
HepG2
Ctrl
Rego(5μM)
Lef(10μM)
Lef(20μM)
C
HepG2
Rego(μM)
Lef(μM)
DAPI
EDU
Merge
D
HepG2
Ctrl
Lenva(10μM)
Lef(20μM)
Lenva(10μM)+Lef(20μM)
**
E
HepG2
Ctrl
Lenva(10μM)
F
HepG2
Lenva(μM)
Lef(μM)
G
L02
Ctrl
Lef(5μM)
Lef(10μM)
Lef(20μM)
ns
H
L02
I
L02
PI
Annexin V-FITC
Apoptotic cells(%)
J
HepG2
Relative mRNA level(%)
Ctrl
Sora(5μM)
Lef(10μM)
Sora(5μM)+Lef(10μM)
Lef(20μM)
Sora(5μM)+Lef(20μM)
Oct4
EpCAM
CD24
K
HepG2-derived xenografts
Mouse weight(g)
Treatment days
Vehicle
Sora(30mg/kg)
Lef(25mg/kg)
Sora(30mg/kg)+Lef(25mg/kg)
Lef(50mg/kg)
Sora(30mg/kg)+Lef(50mg/kg)
L
Heart
Liver
Spleen
Lung
Kidney
Vehicle
Sora (30mg/kg)
Lef (25mg/kg)
Lef (50mg/kg)
Sora(30mg/kg) + Lef(25mg/kg)
Sora(30mg/kg) + Lef(50mg/kg)
M
Plasma ALT(U/L)
Plasma AST(U/L)
Plasma BUN(mmol/L)
Plasma CRE(μmol/L)

Figure S2. Combination of lefamulin and sorafenib significantly inhibits HCC growth in vitro and in vivo.

A-C) HepG2 cells were treated with various concentrations of lefamulin, 5 μM regorafenib alone, or the combination for indicated time, and cell proliferation was evaluated by CCK-8 assay (A) (n = 3), colony formation assay (B), and EDU incorporation assay (C). **D-F**) HepG2 cells were treated with various concentrations of lefamulin, 10 μM lenvatinib alone, or the combination for indicated time, and cell proliferation was evaluated by CCK-8 assay (D) (n = 3), colony formation assay (E), and EDU incorporation assay (F). **G-H**) L02 cells were treated with various concentrations of lefamulin for indicated time, and cell proliferation were evaluated by CCK-8 assay (G) (n = 3), and colony formation assay (H). **I**) L02 cells were treated with various concentrations of lefamulin for 48 hours, and apoptosis was analyzed by flow cytometry (n = 3). **J**) RT-qPCR was carried out to examine mRNA expression of *Oct4*, *EpCAM* and *CD24* in HepG2 cells treated with various concentrations of lefamulin and 5 μM sorafenib separately or in combination for 48 hours (n = 3). **K**) Body weights of mice were monitored upon drug administration (n = 6). **L**) Representative images of H&E staining in mouse organ tissues. Scale bar, 100 μm. **M**) Plasma ALT, AST, BUN and CRE levels were measured in the indicated groups (n = 6). Data are presented as mean ± SEM. t test, $^{*}P < 0.05$, $^{**}P < 0.01$, $^{***}P < 0.001$, and n.s., not significant.

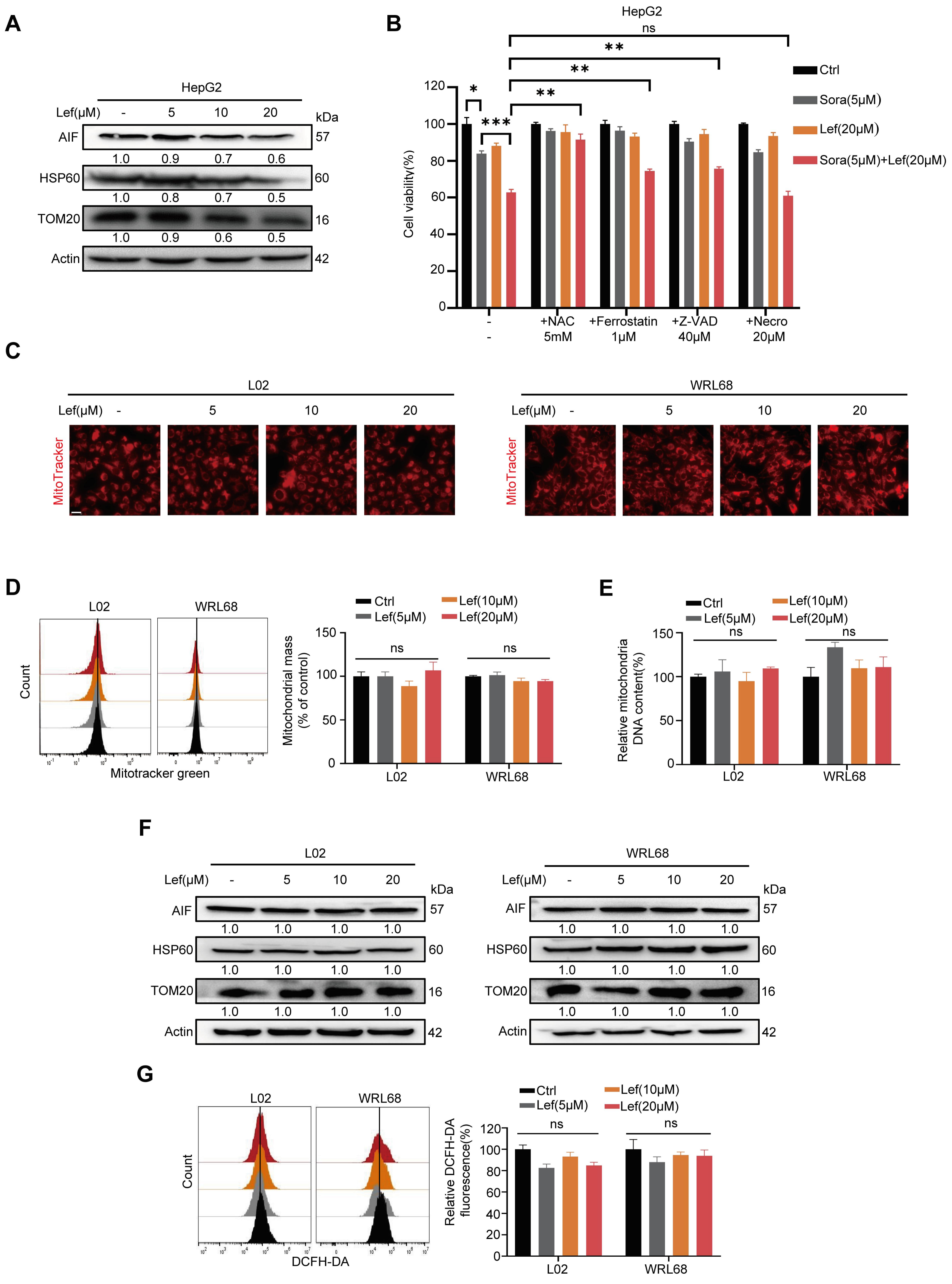
A
HepG2
Lef(μM) - 5 10 20
kDa
AIF 57
1.0 0.9 0.7 0.6
HSP60 60
1.0 0.8 0.7 0.5
TOM20 16
1.0 0.9 0.6 0.5
Actin 42
B
HepG2
ns
**
**
**
*
***
Ctrl
Sora(5μM)
Lef(20μM)
Sora(5μM)+Lef(20μM)
Cell viability(%)
+NAC 5mM
+Ferrostatin 1μM
+Z-VAD 40μM
+Necro 20μM
C
L02
WRL68
Lef(μM) - 5 10 20
MitoTracker
D
L02
WRL68
Count
Mitotracker green
Ctrl
Lef(5μM)
Lef(10μM)
Lef(20μM)
Mitochondrial mass (% of control)
ns
E
Relative mitochondria DNA content(%)
F
L02
WRL68
Lef(μM) - 5 10 20
AIF 57
HSP60 60
TOM20 16
Actin 42
1.0 1.0 1.0 1.0
G
DCFH-DA
Relative DCFH-DA fluorescence(%)

Figure S3. Lefamulin-mediated mitochondrial dysfunction augmented the susceptibility of HCC cells to sorafenib.

A) Immunoblotting of AIF, HSP60 and TOM20 in HepG2 cells treated with various concentrations of lefamulin for 48 hours. **B**) HepG2 cells were treated with sorafenib (5 μM) and/or lefamulin (20 μM) in the presence or absence of pretreatment with different inhibitors for 24 hours, the cell viability was measured by CCK-8 assay, these inhibitors include NAC (an ROS scavenger, 5 mM), Ferrostatin-1 (a ferroptosis inhibitor, 1 μM), Z-VAD-FMK (a apoptosis inhibitor, 40 μM) and Necrosulfonamide (a necroptosis inhibitor, 20 μM) (n = 3). **C**) Representative IF images of mitochondrial morphology in L02 and WRL68 cells treated with various concentrations of lefamulin for 48 hours. Scale bar, 20 μm. **D**) Mitochondrial mass was estimated with MitoTracker Green using flow cytometry in L02 and WRL68 cells treated with various concentrations of lefamulin for 48 hours (n = 3). **E**) RT-qPCR analysis of mitochondrial DNA content in L02 and WRL68 cells treated with various concentrations of lefamulin for 48 hours (n = 3). **F**) Immunoblotting of AIF, HSP60 and TOM20 in L02 and WRL68 cells treated with various concentrations of lefamulin for 48 hours. **G**) Flow cytometric analysis of ROS accumulation using a DCFH-DA probe in L02 and WRL68 cells treated with various concentrations of lefamulin for 48 hours (n = 3). Data are presented as mean ± SEM. t test, *P < 0.05, **P < 0.01, ***P < 0.001, and n.s., not significant.

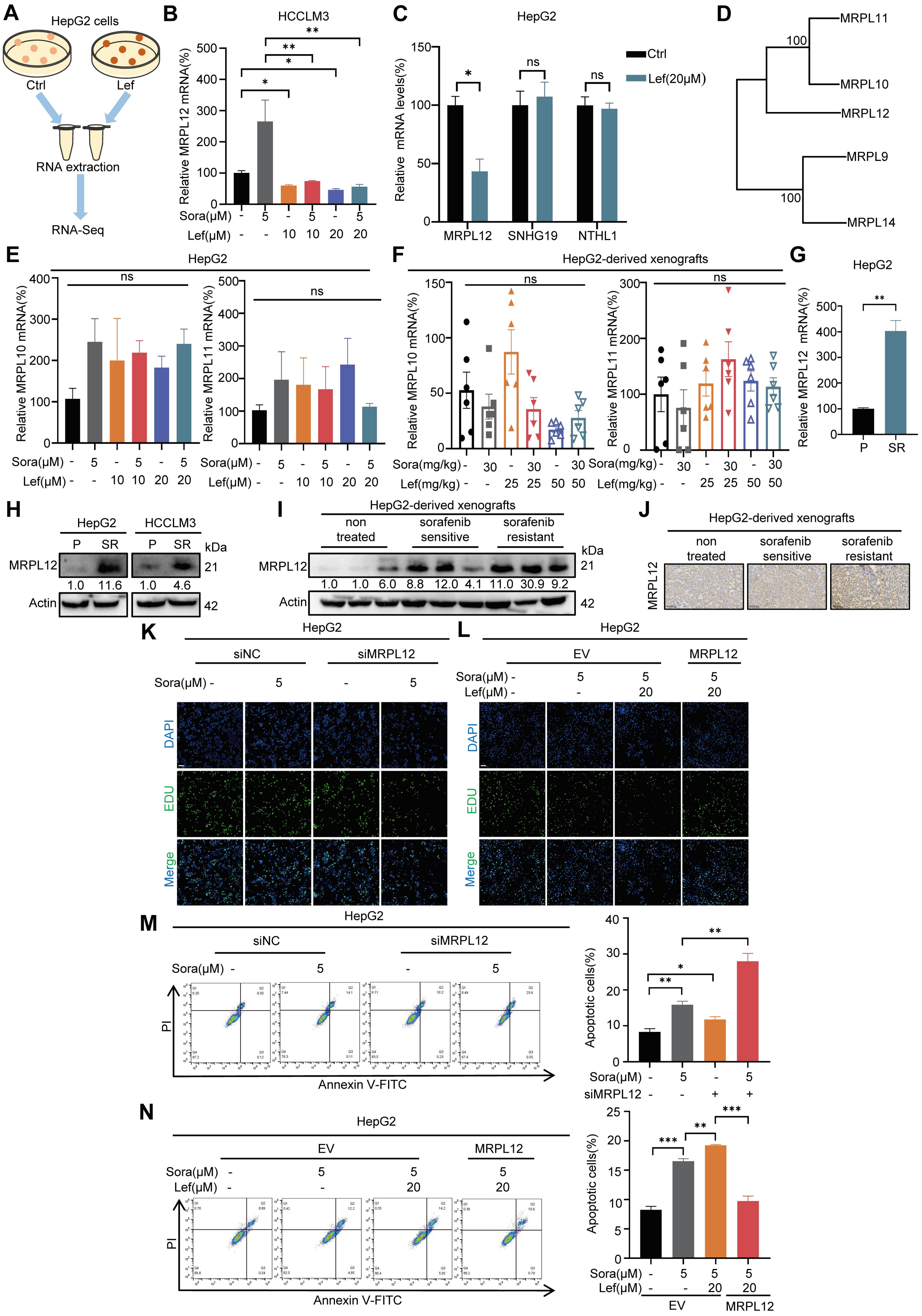
A
HepG2 cells
Ctrl
Lef
RNA extraction
RNA-Seq
B
HCCLM3
Relative MRPL12 mRNA(%)
Sora(μM)
Lef(μM)
C
HepG2
Relative mRNA levels(%)
Ctrl
Lef(20μM)
MRPL12
SNHG19
NTHL1
D
MRPL11
MRPL10
MRPL12
MRPL9
MRPL14
E
HepG2
Relative MRPL10 mRNA(%)
Relative MRPL11 mRNA(%)
F
HepG2-derived xenografts
Sora(mg/kg)
Lef(mg/kg)
G
HepG2
Relative MRPL12 mRNA(%)
P
SR
H
HepG2
HCCLM3
MRPL12
Actin
kDa
I
HepG2-derived xenografts
non treated
sorafenib sensitive
sorafenib resistant
J
K
siNC
siMRPL12
DAPI
EDU
Merge
L
EV
MRPL12
M
PI
Annexin V-FITC
Apoptotic cells(%)
N

Figure S4. Lefamulin impairs mitochondrial metabolism via downregulation of MRPL12.

A) Scheme illustrating of differentially expressed genes of lefamulin or DMSO treated HepG2 cells. **B**) RT-qPCR was carried out to examine mRNA expression of *MRPL12* in HCCLM3 cells treated with lefamulin and/or sorafenib (n = 3). **C**) RT-qPCR was carried out to examine mRNA expression of *MRPL12*, *SNHG19* and *NTHL1* in HepG2 cells treated with lefamulin (n = 3). **D**) Homologous analysis of MRPL12 within mitochondrial ribosomal proteins. **E**,**F**) RT-qPCR was carried out to examine mRNA expression of *MRPL10*, *MRPL11* in HepG2 cells (E) (n = 3) and HepG2-derived xenografts (F) (n = 6) treated with lefamulin and/or sorafenib. **G**,**H**) RT-qPCR (G) (n = 3) and western blot (H) were carried out to examine mRNA and protein expression of MRPL12 in sensitive and resistant HepG2 and HCCLM3 cells. **I**) Western blot was carried out to examine protein expression of MRPL12 in sorafenib-sensitive and sorafenib-resistant HepG2-derived xenografts. **J**) Representative images of IHC staining of MRPL12 of sorafenib-sensitive and sorafenib-resistant HepG2-derived xenografts. Scale bar, 50 µm. **K**,**L**) Effect of MRPL12 knockdown (K) or overexpression (L) on proliferation in HepG2 cells measured by EDU incorporation assay. Scale bar, 100 µm. **M**,**N**) Effect of MRPL12 knockdown (M) or overexpression (N) on apoptosis in HepG2 cells measured by flow cytometry assay (n = 3). Data are presented as mean ± SEM. t test, *P < 0.05, **P < 0.01, ***P < 0.001, and n.s., not significant.

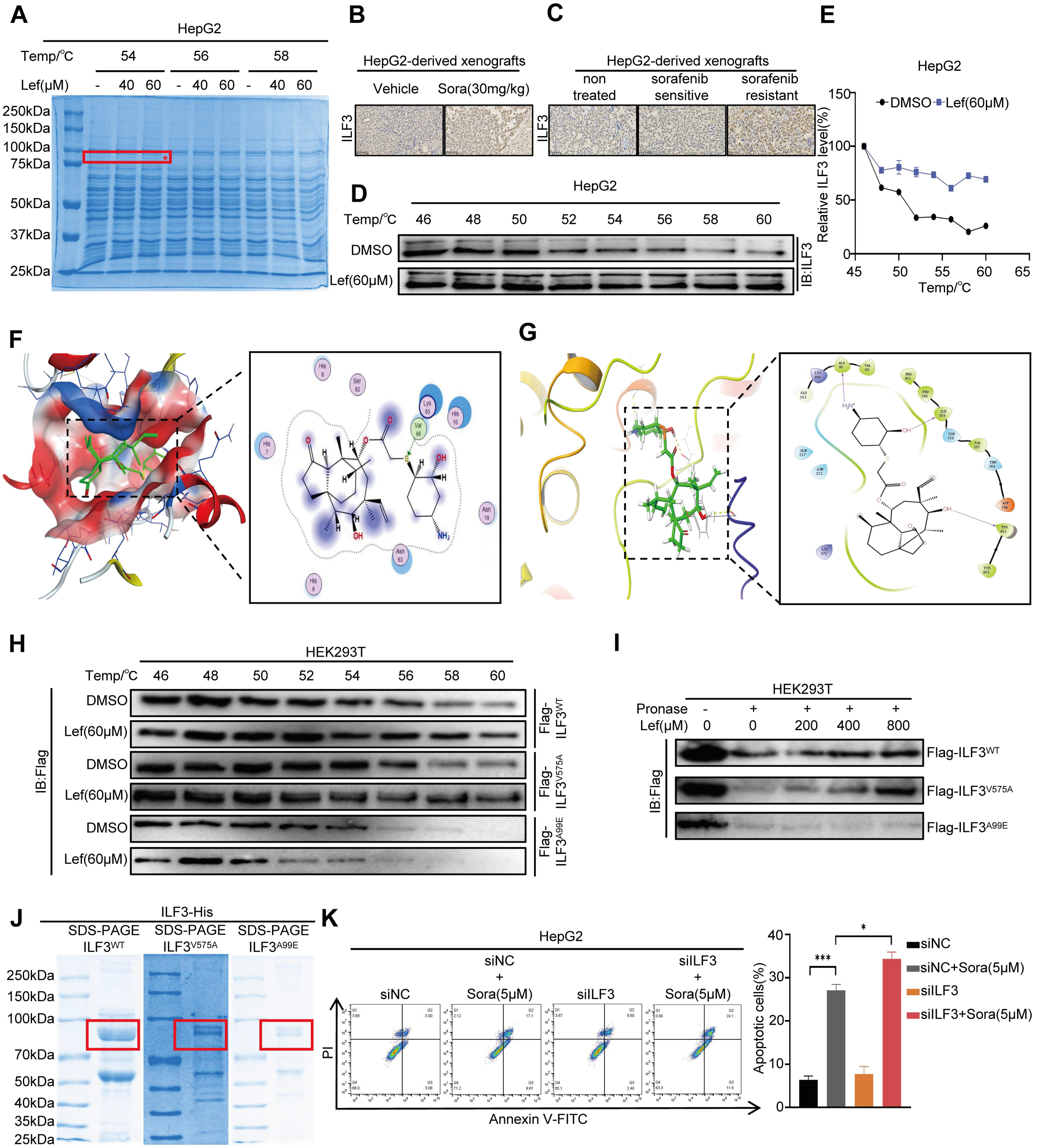
A
HepG2
Temp/°C
54
56
58
Lef(μM)
- 40 60 - 40 60 - 40 60
250kDa
150kDa
100kDa
75kDa
50kDa
37kDa
25kDa
B
HepG2-derived xenografts
Vehicle
Sora(30mg/kg)
ILF3
C
HepG2-derived xenografts
non treated
sorafenib sensitive
sorafenib resistant
ILF3
D
HepG2
Temp/°C
46 48 50 52 54 56 58 60
DMSO
Lef(60μM)
IB:ILF3
E
HepG2
DMSO
Lef(60μM)
Relative ILF3 level(%)
Temp/°C
F
G
H
HEK293T
Temp/°C
46 48 50 52 54 56 58 60
DMSO
Lef(60μM)
Flag-ILF3WT
Flag-ILF3V575A
Flag-ILF3A99E
IB:Flag
I
HEK293T
Pronase
Lef(μM)
0 0 200 400 800
IB:Flag
Flag-ILF3WT
Flag-ILF3V575A
Flag-ILF3A99E
J
ILF3-His
SDS-PAGE ILF3WT
SDS-PAGE ILF3V575A
SDS-PAGE ILF3A99E
250kDa
150kDa
100kDa
70kDa
50kDa
40kDa
35kDa
25kDa
K
HepG2
siNC
siNC + Sora(5μM)
siILF3
siILF3 + Sora(5μM)
PI
Annexin V-FITC
Apoptotic cells(%)
***
*
siNC
siNC+Sora(5μM)
siILF3
siILF3+Sora(5μM)

Figure S5. Lefamulin targets ILF3 to sensitize HCC to sorafenib.

A) The differential band between lefamulin- and DMSO- treated HepG2 cells identified by SDS-PAGE and Coomassie blue staining. **B**) Representative images of IHC staining of ILF3 of tumors from nude mice. Scale bar, 50 μm. **C**) Representative images of IHC staining of ILF3 of HepG2-derived sorafenib-sensitive and sorafenib-resistant xenografts. Scale bar, 50 μm. **D**,**E**) CETSA was used to evaluate the binding of lefamulin with ILF3. The expression of ILF3 was detected by western blot (n = 3). **F**,**G**) Representative images of auto-docking between lefamulin and ILF3, the solution NMR and crystal structure were obtained from PDB (2L33, score = -7) (F) and AlphaFold (AF-Q12906-F1, score = -5.074) (G), respectively. **H**,**I**) CETSA (H) and DARTS (I) were used to evaluate the binding site of lefamulin with ILF3. The expression of Flag-ILF3 was detected by western blot. **J**) SDS-PAGE indicating the purification of the $ILF3^{WT}$, $ILF3^{A99E}$, $ILF3^{V575A}$ protein. **K**) Effect of ILF3 knockdown and/or sorafenib treatment on apoptosis in HepG2 cells measured by flow cytometry assay (n = 3). Data are presented as mean ± SEM. t test, *P < 0.05 and ***P < 0.001.

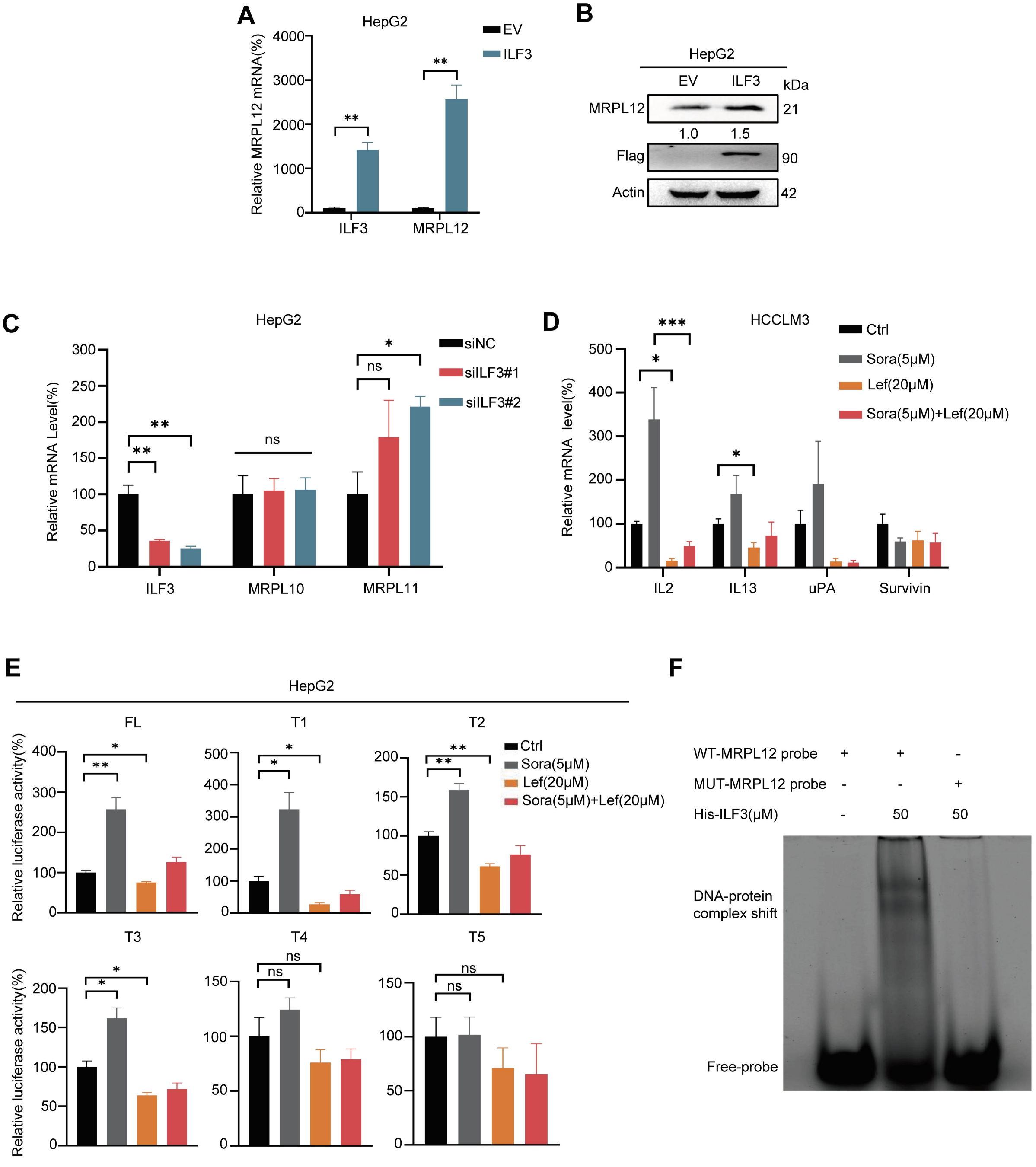
A
HepG2
Relative MRPL12 mRNA(%)
EV
ILF3
ILF3
MRPL12
B
HepG2
EV
ILF3
kDa
MRPL12
21
1.0
1.5
Flag
90
Actin
42
C
HepG2
Relative mRNA Level(%)
siNC
siILF3#1
siILF3#2
ILF3
MRPL10
MRPL11
D
HCCLM3
Relative mRNA level(%)
Ctrl
Sora(5μM)
Lef(20μM)
Sora(5μM)+Lef(20μM)
IL2
IL13
uPA
Survivin
E
HepG2
FL
T1
T2
T3
T4
T5
Relative luciferase activity(%)
Ctrl
Sora(5μM)
Lef(20μM)
Sora(5μM)+Lef(20μM)
F
WT-MRPL12 probe
MUT-MRPL12 probe
His-ILF3(μM)
DNA-protein complex shift
Free-probe

Figure S6. ILF3 is a transcriptional activator of MRPL12.

A,B) The mRNA (A) (n = 3) and protein (B) expression of MRPL12 in HepG2 cells with or without ILF3 overexpression. **C**)The mRNA expression of *MRPL10*, *MRPL11* in HepG2 cells with or without ILF3 knockdown (n = 3). **D**) RT-qPCR was carried out to examine mRNA expression of ILF3 downstream target genes, including *IL2*, *IL13*, *uPA* and *Survivin* in HCCLM3 cells treated with lefamulin and/or sorafenib (n = 3). **E**) Fold change in luciferase activity driven by MRPL12-promoter reporter under lefamulin and/or sorafenib treatment in HepG2 cells (n = 3). **F**) EMSA experiment with 25-bp MRPL12 promoter oligonucleotides and 50 μM ILF3 protein. Data are presented as mean ± SEM. t test, *P < 0.05, **P < 0.01, ***P < 0.001, and n.s., not significant.

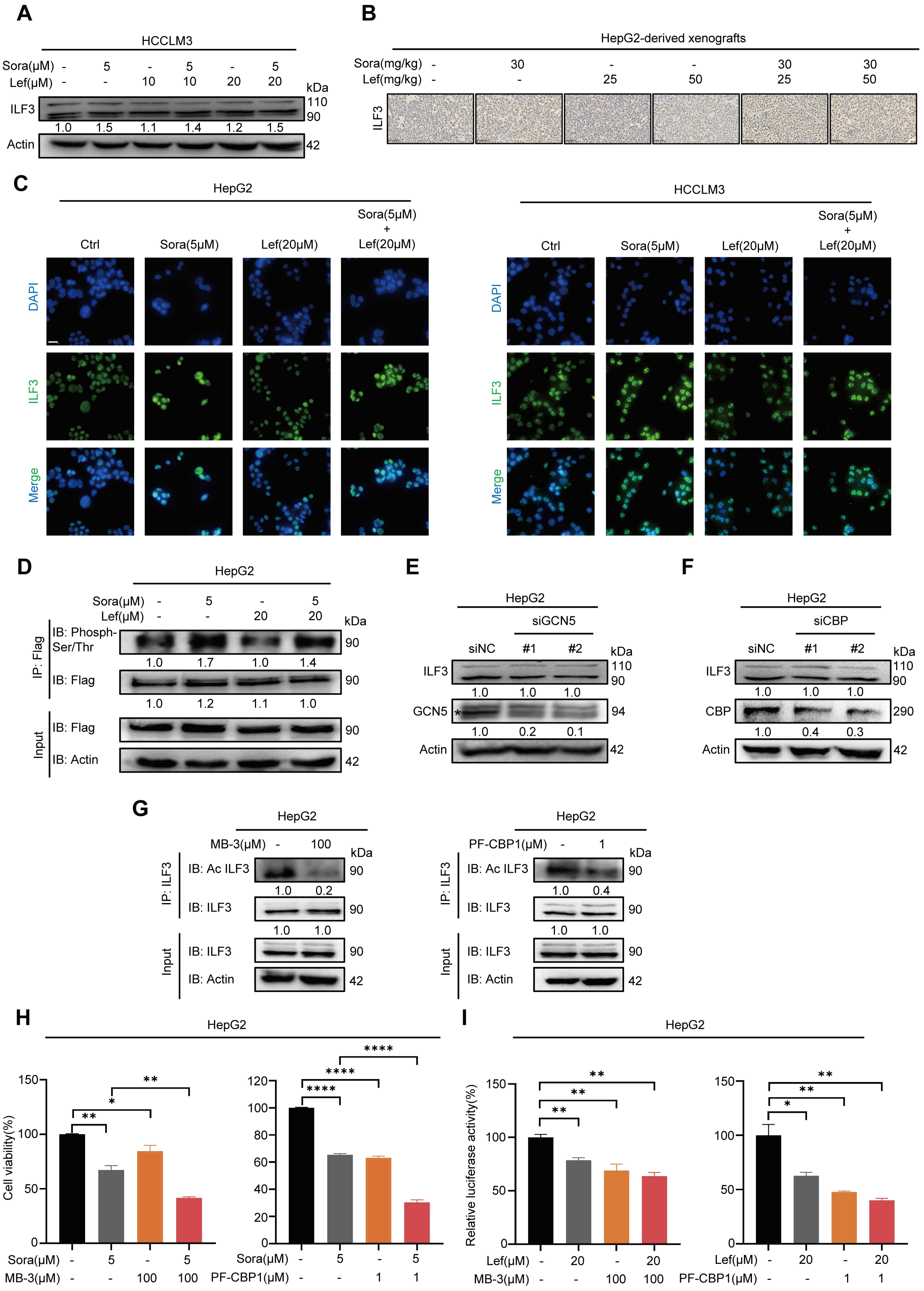
A
HCCLM3
Sora(μM)
Lef(μM)
ILF3
Actin
kDa
110
90
42
1.0 1.5 1.1 1.4 1.2 1.5
B
HepG2-derived xenografts
Sora(mg/kg)
Lef(mg/kg)
ILF3
C
HepG2
HCCLM3
Ctrl
Sora(5μM)
Lef(20μM)
Sora(5μM) + Lef(20μM)
DAPI
ILF3
Merge
D
HepG2
IP: Flag
Input
IB: Phosph-Ser/Thr
IB: Flag
IB: Actin
1.0 1.7 1.0 1.4
1.0 1.2 1.1 1.0
E
HepG2
siGCN5
siNC #1 #2
GCN5
1.0 0.2 0.1
94
F
siCBP
CBP
1.0 0.4 0.3
290
G
MB-3(μM)
PF-CBP1(μM)
IP: ILF3
IB: Ac ILF3
IB: ILF3
1.0 0.2
1.0 0.4
H
Cell viability(%)
I
Relative luciferase activity(%)

Figure S7. Lefamulin inhibits GCN5/CBP-mediated acetylation of ILF3 and subsequent transcriptional activation.

A) Western blot was carried out to examine protein expression of ILF3 in HCCLM3 cells treated with lefamulin and/or sorafenib. **B**) Representative images of IHC staining of ILF3 of tumors from nude mice. Scale bar, 50 μm. **C**) Representative images of intracellular distribution of ILF3 in HepG2 and HCCLM3 cells treated with 5 μM and/or 20 μM lefamulin. Scale bar, 20 μm. **D**) Western blot was carried out to examine the expression of phosphorylated ILF3 in HepG2 cells transfected with ILF3-Flag. **E**,**F**) Western blot was carried out to examine protein expression of ILF3 in HepG2 cells after GCN5 (E) or CBP (F) knockdown. **G**) Western blot was carried out to examine the expression of acetylated ILF3 in HepG2 cells in the presence or absence of 100 μM MB-3/1 μM PF-CBP1. **H**) Cell viability was examined in HepG2 cells treated with 100 μM MB-3/1 μM PF-CBP1 and/or 5 μM sorafenib by CCK-8 assay (n = 3). **I**) Luciferase activity was examined in HepG2 cells treated with 100 μM MB-3/1 μM PF-CBP1 and/or 20 μM lefamulin by dual luciferase analysis kit (n = 3). Data are presented as mean ± SEM. t test, $^*P < 0.05$, $^{**}P < 0.01$, and $^{****}P < 0.0001$.

Supplementary tables

Table S1. The cell viability of 32 candidate compounds treatment in HepG2 SR cells, related to Figure 1B.

Name	Cell viability (sorafenib+drugs *vs* sorafenib) %	IC50 (drugs) µM	Is there any related anti-tumor research
Zinc Pyrithione	15.37	8.14	Yes
Ganciclovir	18.42	62.79	Yes
Valnemulin HCl	23.75	>100	No
Vorinostat	31.16	61.81	Yes
Foretinib	32.05	>100	Yes
Retapamulin	34.87	>100	No
Disulfiram	35.11	0.38	Yes
Valaciclovir HCl	35.59	>100	Yes
Formestane	40.92	>100	Yes
Mitoxantrone HCl	42.57	13.72	Yes
Mitomycin C	44.07	50.26	Yes
Deflazacort	45.40	>100	No
Entinostat	46.30	45.61	Yes
Uprosertib	46.30	>100	Yes
Osimertinib	48.87	76.68	Yes
Dasatinib	49.10	>100	Yes
Methenamine	49.27	>100	Yes
Sulfadiazine	51.67	56.16	Yes
Ganetespib	52.03	>100	Yes
Decitabine	53.16	>100	Yes
Rociletinib	54.17	>100	Yes
Diclofenac Sodium	55.64	>100	Yes
Vandetanib	56.05	>100	Yes
Ulixertinib	56.1	>100	Yes
Rigosertib	56.47	>100	Yes
Navitoclax	56.81	>100	Yes
Curcumin	56.84	>100	Yes
Afatinib	57.74	>100	Yes
Roxithromycin	57.85	>100	No
Ceritinib	58.22	25.03	Yes
Nintedanib	58.92	51.04	Yes
Idasanutlin	58.93	76.08	Yes

Table S2. The list of 27 potential target proteins of lefamulin (75kDa<MW<100kDa), related to Figure 5A.

Name	Abundances
HSP90AB1	101047647.6
VCP	41667423.76
EL52	39829939.87
ILF3	29730947.8
PFKP	13248060.0234375
PYGB	11772516.7578125
EEF2	9370426.6328125
ADD3	7264347.3125
GPHN	7083090.453125
HSP90B1	6424425.546875
CTNNB1	5360098.125
SSRP1	3864963.890625
CUL5	2750284.3203125
CUL4B	2269490.4375
PHACTR4	2225136.5546875
H6PD	1046612.296875
UBA2/WTIP fusion protein	966279.140625
DNAJC10	862265.671875
CSDE1	849912.7265625
ElaC homolog protein 2	760869.4296875
P3H3	519716.5703125
CAND1	482191.921875
PDXDC1	396467.5546875
VAC14	305881.703125
DDHD2	287444.0859375
SEMA4G	206113.5390625
HNRNPU	150755.5625

Table S3. Primers and siRNA sequences used in this study, related to Supplementary materials and Methods.

**Primers used for RT-qPCR**		
**Genes**	**Forward sequence(5'-3')**	**Reverse sequence(5'-3')**
Human		
GAPDH	GACCTGCCGTCTAGAAAAAC	TTGAAGTCAGAGGAGACCAC
ILF3	CCTGACAAAGCACGGCAAGAAC	CCAGCACCTTGGAACTTCTGTC
MRPL12	ATCCCCATAGCGAAAGAACGG	GGACGAGGTTGATGCCTTGG
MRPL10	CACCGTCGTGTGATGCACTT	CGGCTATCATTCGGTTGTCCT
MRPL11	GGCGTTTCCATCAACCAGTTT	TGCGGGCAATCTCATACACAT
MDR1	TTGCTGCTTACATTCAGGTTTCA	AGCCTATCTCCTGTCGCATTA
BCRP	CAGGTGGAGGCAAATCTTCGT	ACCCTGTTAATCCGTTCGTTTT
MRP1	CTCTATCTCTCCCGACATGACC	AGCAGACGATCCACAGCAAAA
Oct4	CTGGGTTGATCCTCGGACCT	CCATCGGAGTTGCTCTCCA
EpCAM	TGATCCTGACTGCGATGAGAG	CTTGTCTGTTCTTCTGACCCC
CD24	CTCCTACCCACGCAGATTTATTC	AGAGTGAGACCACGAAGAGAC
IL2	AACTCCTGTCTTGCATTGCAC	GCTCCAGTTGTAGCTGTGTTT
IL13	CCTCATGGCGCTTTTGTTGAC	TCTGGTTCTGGGTGATGTTGA
uPA	GGGAATGGTCACTTTTACCGAG	GGGCATGGTACGTTTGCTG
Survivin	CTGCACACCTGACAAGATGG	CAGCCTCTCTTTCTCCATGC
D-loop2	GGCTCTCAACTCCAGCATGT	AGGACGAGGGAGGCTACAAT
G6PC	CTGTCTTTGATTCCTGCCTCAT	GTGGCTGTGCAGACATTCAA
SNHG19	AACATGAGGGAATGAATGAG	TAGACCAAAACAGAAGGAAC
NTHL1	CGCGGAAAGCACAGAGACT	CTCATGGCACGGATGTTGAC

**Primers used for ChIP-qPCR**		
**Gene**	**Forward sequence(5'-3')**	**Reverse sequence(5'-3')**
ILF3-site (-800 to -300bp)	GGTTCATGTGTGCCCTCCG	CCGCAGTAATCCACGTCTCC

**Primers for plasmids construction**		
**Genes**	**Forward sequence(5'-3')**	**Reverse sequence(5'-3')**
pCMV-N-Myc -*MRPL12*	GATCTGAGCCCGGGCGGATCC ATGCTGCCGGCGGCCGCTCG	TCTGTCGACGATATCGAATTC CTCCAGAACCACGGTGCCGC
pCMV-C-Flag -*ILF3*	CGCTCTAGCCCGGGCGGATCC ATGCGTCCAATGCGAATTTT	TCTGTCGACGATATCGAATTC TCTGTACTGGTAGTTCATGC
pET28a-His-*IL*	CAGCAAATGGGTCGCGGATCC	TTGTCGACGGAGCTCGAATTC

F3	ATGCGTCCAATGCGAATTTT	TCTGTACTGGTAGTTCATGC
pGL4-luc-*MRPL12*-FL	CTGGCCTAACTGGCCGGTACCGCATGAGCTGGCTGCATGAG	CCGAGGCCAGATCTTGATATCGCGGAACGCAGCGGCCCGAA
pGL4-luc-*MRPL12*-T1	CTGGCCTAACTGGCCGGTACCCCCAGCCCTGTGGGTCATGG	CCGAGGCCAGATCTTGATATCGCGGAACGCAGCGGCCCGAA
pGL4-luc-*MRPL12*-T2	CTGGCCTAACTGGCCGGTACCATCCACCTGCCTCGGGCTCC	CCGAGGCCAGATCTTGATATCGCGGAACGCAGCGGCCCGAA
pGL4-luc-*MRPL12*-T3	CTGGCCTAACTGGCCGGTACCTGTGGAGAGACGGGCAGGAT	CCGAGGCCAGATCTTGATATCGCGGAACGCAGCGGCCCGAA
pGL4-luc-*MRPL12*-T4	CTGGCCTAACTGGCCGGTACCCGGCACTGGGGGCCCGGAGC	CCGAGGCCAGATCTTGATATCGCGGAACGCAGCGGCCCGAA
pGL4-luc-*MRPL12*-T5	CTGGCCTAACTGGCCGGTACCTCTAGCTGCCGCCACAGACG	CCGAGGCCAGATCTTGATATCGCGGAACGCAGCGGCCCGAA

**siRNA sequences for knockdown**	
**Genes**	**siRNA sequence(5'-3')**
si*MRPL12*#1	AACGTTGAAGATCCAGGAT
si*MRPL12*#2	UCAAGAACUACAUCCAAGGCAUCAA
si*ILF3*#1	CCATGTCTGTGGAGGTTGA
si*ILF3*#2	CAGGCAGAGTCCGATAACA
si*GCN5*#1	CGTGCTGTCACCTCGAATGA
si*GCN5*#2	TCATGTCTGTTCACAAGGAA
si*CBP*#1	ATCGCCACGTCCCTTAGTAAC
si*CBP*#2	GGGATGAATATTATCACTTAT

Table S4. Antibodies used in this study, related to Supplementary materials and Methods.

Antibody	Source	Catalog number	Application
Actin	Proteintech	No. 66009-1-Ig	WB: 1:1000
ILF3	Proteintech	No. 19887-1-AP	WB: 1:2000<br>IF: 1:100<br>IHC: 1:100
MRPL12	Proteintech	No.14795-1-AP	WB: 1:1000<br>IHC: 1:250
GCN5	Proteintech	No. 66575-1-Ig	WB: 1:2000
CBP	Proteintech	No. 22277-1-AP	WB: 1:1000
Myc-tag	Cell Signaling Technology	No. 2278	WB: 1:1000
Flag-tag	Proteintech	No. 66008-4-Ig	WB: 1:5000<br>IP: 2 μg
Ki67	Proteintech	No. 27309-1-AP	IHC: 1:2000
Acetylated-Lysine	Cell Signaling Technology	No. 9441	WB: 1:1000
Phospho-(Ser/Thr) Phe	Cell Signaling Technology	No. 9631	WB: 1:1000
HSP60	Proteintech	No.15282-1-AP	WB: 1:2000
TOM20	Proteintech	No. 11802-1-AP	WB: 1:2000
AIF	Proteintech	No. 17984-1-AP	WB: 1:2000

Data file S1. The list of 1430 FDA-approved compound library.

Data file S2. Summary of mass spectrum (MS) analysis of potential lefamulin-binding proteins, related to Figure 5A.

Data file S3. Raw data for main figures and supplementary figures.
